# Topology Assisted Clustering of Temporal fMRI Brain Networks With Use-Case in Mitigating Non-Neural Multi-Site Variability

**DOI:** 10.1109/access.2025.3616256

**Published:** 2025-09-30

**Authors:** AHMEDUR RAHMAN SHOVON, SIDHARTH KUMAR, GOPIKRISHNA DESHPANDE

**Affiliations:** 1 Department of Computer Science, University of Illinois Chicago, Chicago, IL 60607, USA; 2 Auburn University Neuroimaging Center, Department of Electrical and Computer Engineering, Auburn University, Auburn, AL 36849, USA; 3 Department of Psychological Sciences, Auburn University, Auburn, AL 36849, USA; 4 Alabama Advanced Imaging Consortium, Birmingham, AL 35294, USA; 5 Center for Neuroscience, Auburn University, Auburn, AL 36849, USA; 6 Department of Heritage Science and Technology, Indian Institute of Technology Hyderabad, Hyderabad 502285, India; 7 Department of Psychiatry, National Institute of Mental Health and Neurosciences, Bengaluru 560030, India

**Keywords:** Temporal fMRI, topological data analysis, persistent homology

## Abstract

Using temporal analysis of fMRI (functional Magnetic Resonance Imaging) data, we can characterize dynamic changes in brain connectivity over time. However, dynamic temporal analysis of fMRI data is challenging due to the high dimensionality of the datasets. Another fundamental challenge of dynamic temporal analysis of fMRI is the presence of non-neural artifacts that add sources of variation in the data that are not directly related to brain activity. For example, when data are acquired at different scanners at different temporal sampling rates and later analyzed as a single dataset, we have to contend with different number of image snapshots for different subjects. Also, high-frequency scans lead to more fine-grained temporal snapshotting than low-frequency scans. These factors can obscure true neural signals and lead to inconsistent characterization of dynamic brain connectivity across scans. Existing graph-based solutions often struggle with parameter sensitivity, since their outcomes depend heavily on selecting an arbitrary correlation threshold for defining network edges. In contrast, topological data analysis (TDA) sweeps across all threshold values to track the persistence of connectivity features, making it more robust for capturing fine-grained temporal dynamics. Clustering methods become imperative in this context as they offer a powerful means to uncover underlying structures within the high-dimensional temporal data. We address these challenges by developing a topological data analysis based temporal clustering pipeline targeted for dynamic functional connectivity derived from fMRI datasets that can preserve the dynamics of the temporal datasets and mask out the non-neural variability induced by varying sampling rates. The TDA-based pipeline extracts robust features that are invariant to non-neural noise and uses them to perform temporal clustering. We evaluate our framework by performing temporal clustering of resting-state fMRI-derived dynamic functional connectivity brain networks obtained from 316 subjects, each of whom was scanned thrice using different temporal sampling periods. The efficacy of our TDA-based pipeline is compared against three alternative approaches: direct time-series clustering, PCA-based dimensionality reduction and clustering, and a traditional fully connected network analysis pipeline with MDS-based dimensionality reduction. Additionally, we demonstrate that for a majority of cases, the number of clusters remains consistent for the same subjects scanned at different temporal sampling rates– showcasing the greater robustness of our TDA-based pipeline compared to other pipelines. The TDA pipeline achieved higher overlaps (59 %) in optimal cluster numbers across sampling cohorts, as well as higher pairwise similarity (74–77 %) between subjects’ cluster solutions. This indicates that incorporating network topology via TDA enables more robust clustering of temporal fMRI datasets despite changes in sampling rates.Furthermore, we validate our method on a clinical dataset (ADHD-200). The TDA-based pipeline successfully captures consistent clustering patterns across different sites and scanning protocols, with higher stability of cluster assignments (> 80% similarity) and better separation of subject-level dynamics compared to existing approaches. This reinforces the method’s robustness in multisite, multi-condition settings. Our results demonstrate that incorporating network topology via TDA significantly enhances the reliability of temporal clustering in fMRI studies, offering a robust framework for studying brain dynamics across heterogeneous acquisition settings.

## I. INTRODUCTION

Temporal fMRI (functional Magnetic Resonance Imaging) analysis [1], [2] is a technique used to study brain activity over time, with a focus on capturing changes in neural activity as they unfold during various cognitive tasks or in resting state conditions. Resting-state functional MRI (rs-fMRI), in particular, examines spontaneous fluctuations in brain activity that occur when a subject is at rest and not performing a specific task. This approach aims to identify intrinsic functional connectivity patterns within the brain, which can reveal networks and interactions that are present even in the absence of external stimuli. Temporal resting-state fMRI analysis is crucial for understanding how the brain processes information dynamically and how the interactions between different brain regions change with time, referred to as dynamic functional connectivity (DFC). It has been shown that DFC is very important for characterizing the healthy brain [3], [4], as well as in various brain disorders [5], [6], [7].

Temporal fMRI data typically has a temporal resolution on the order of hundreds of milliseconds to a couple of seconds, with each time point representing a snapshot of brain activity at a particular moment during the scan. DFC calculated from rs-fMRI data sampled at different rates tends to capture underlying dynamics evolving at different scales. Also, rs-fMRI data with higher sampling frequency tends to have more time points compared to that sampled at lower frequency for the same experimental duration. More time points tend to increase the robustness of DFC estimates. Given these factors, DFC calculated from rs-fMRI data sampled at different frequencies are not comparable. This is problematic since different groups tend to have different sampling rates owing to other factors, such as the capabilities of the MRI scanner as well as SNR available for a given field strength and field of view. Given the ever-increasing demands on sample size, pooling data acquired from multiple sites has become a priority, but that seems impractical as data across sites is not acquired using the same sampling frequency. Here, we seek to address this problem. Our reasoning is that a video captured at two different frame rates must convey the same content, even though they might not be of the same quality. Our quest is to devise a method that would capture the underlying content correctly, irrespective of how fast or slow the temporal dynamics are sampled. Obviously, DFC is not doing this job. Instead, we propose to use topological data analysis (TDA), which has been shown to characterize the structure underlying the data, which may be invariant to how the data is acquired [8].

Within the domain of functional brain imaging research, there is a growing trend towards the adoption of topological data analysis (TDA), an algebraic topology-based mathematical approach [9]. More recently, topological data analysis tools such as persistent homology (PH) have been utilized to study complex networks [10] including fMRI network dynamics [11], [12], [13], [14]. The persistence of topological features over a range of spatial scales provides insight into the robustness and stability of network architecture. Persistent homology generates topological barcodes that serve as a quantitative fingerprint for complex networks. Specifically, persistent homology tracks the emergence and disappearance of topological features such as connected components, loops, and higher-dimensional cavities across a range of thresholds applied to the network. Importantly, statistical distances between barcodes, such as the Wasserstein distance(WD) [15], [16], can be computed to quantitatively compare network topologies robustly. Thus, persistent homology barcodes provide a distinctive topological signature and metric for interrogating complex network architecture over the traditional graph-based tools [8]. Recent studies have further extended the use of TDA to unsupervised learning and clustering applications. For example, TDA-based clustering of functional brain networks has been successfully applied to Alzheimer’s disease cohorts, revealing significant associations between functional topology and brain morphometry using Wasserstein distance kernels [17]. In task-based fMRI, TDA pipelines have outperformed traditional vectorization methods in classifying condition-specific activity, highlighting their ability to capture individualized functional profiles [11]. More broadly, TDA-based classifiers have shown promising results across domains like trajectory classification and imbalanced multi-class datasets, underscoring the versatility and power of topological representations in complex data settings [18], [19]. We harness this capability to compare the similarities of temporal dynamics of brain networks extracted from different sampling periods. We develop a TDA-based statistical data processing pipeline targeted for temporal fMRI datasets that preserves the temporal dynamics of rs-fMRI datasets with the ability to mask out the non-neural variability induced by varying temporal sampling rates.

We evaluate the efficacy of our framework using three data cohorts, each of which corresponds to rs-fMRI data acquired for a subject at a different temporal sampling rate. The input to our pipeline comprises functional connectivity networks (FCNs) derived from rs-fMRI data acquired from 316 subjects, scanned at three temporal frequencies: $f_{\mathrm{high}}=645ms,\;f_{\mathrm{medium}}=1400ms$, and $f_{\mathrm{low}}=2500ms$. High temporal frequency ($f_{\mathrm{high}}=645ms$) corresponds to fine-grained snapshotting, yielding a total of 754 time-steps; medium temporal frequency ($f_{\mathrm{medium}}=1400ms$) scan yields 336 time-steps; and on the other end of the spectrum is low-frequency scans of $f_{\mathrm{low}}=2500ms$, which yields 86 time-steps. The total number of adjacency matrices (FCNs) used in our study is therefore 371,616 (= 316 × 754 for $f_{\mathrm{high}}+316\times 336$ for $f_{\mathrm{medium}}+316\times 86$ matrices for $f_{\mathrm{low}}$). In essence, our statistical pipeline deals with a complex high-dimensional space, the three cohorts are each 4-dimensional spaces, of resolution 113 × 113 × *timestep*# × *subjects*#. 113 × 113 corresponds to the spatial resolution of each of the individual FCN (adjacency matrix), capturing the pairwise connectivity strengths among the 113 spatial regions of the brain. The *timestep*# is respectively 754, 336, and 86 for the three temporal frequencies $f_{\mathrm{high}},\;f_{\mathrm{medium}}$, *and*, $f_{\mathrm{low}}$. *subject*# is 316. Demonstrating any notion of similarity across such a high-dimensional space is a challenging task, which is further exacerbated by the fact that the resolution across the three spaces varies (along the *timestep*# dimension). To solve this challenge, we rely on a well-established notion that the resting-state brain typically oscillates between a handful of discrete states. We develop a statistical pipeline that uses persistent homology from TDA to gradually reduce the high-dimensional temporal data to two dimensions – which is then reduced further to a one-dimensional scalar quantity that captures the total number of clusters. The novelty of this paper is twofold: (1) developing a statistical framework based on TDA techniques to effectively reduce the complex high dimensional space into simple 1D space (corresponding to the number of clusters), and (2) demonstrating that the number of temporal clusters of same subjects across the three temporal sampling rates is *indeed* the same, unlike existing methods.

To further evaluate the performance of our TDA pipeline, we developed three alternative statistical data analysis pipelines that utilize the same datasets. These pipelines represent different approaches to data processing and dimensionality reduction that have been used traditionally, allowing for a comprehensive comparison of our TDA pipeline against other established methods.

1. **Direct time-series clustering pipeline**: The first pipeline employs a direct clustering across time, where the original time-series data is reshaped into a suitable format for clustering algorithms. This direct approach bypasses the need for dimensionality reduction, retaining the full temporal dynamics of the data.
2. **PCA-Based dimensionality reduction and clustering pipeline**: The second pipeline utilizes principal component analysis (PCA) to reduce the dimensionality of the time-series data before applying a clustering algorithm. PCA identifies the principal components, which are linear combinations of the original features that capture the maximum variance in the data. By retaining only the most significant principal components, PCA reduces the dimensionality while preserving the essential information for clustering.
3. **Traditional dynamic FCN analysis pipeline with MDS-based dimensionality reduction**: The third pipeline adopts a traditional dynamic FCN (dFCN) analysis approach similar to our TDA pipeline with multidimensional scaling (MDS) for dimensionality reduction. This pipeline calculates the pairwise correlation coefficients between all 113 brain regions at every time point, resulting in a temporally varying correlation matrix. MDS is then applied to the correlation matrix to reduce its dimensionality while preserving the underlying connectivity patterns. This approach retains the inter-subject connectivity information, which differs from the aforementioned pipelines.

To determine the appropriate number of clusters for our TDA and the three nonTDA pipelines, we employ the k-means clustering algorithm in conjunction with the silhouette criterion. Since the number of clusters is insufficient to effectively capture the similarities between different temporal sampling periods, we conduct cluster distance comparisons both across the data cohorts ($f_{\mathrm{high}},\;f_{\mathrm{medium}}$, and $f_{\mathrm{low}}$) and between individual data cohort pairs ($f_{\mathrm{high}}-f_{\mathrm{medium}},\;f_{\mathrm{low}}-f_{\mathrm{medium}}$, and $f_{\mathrm{high}}-f_{\mathrm{low}}$). We further evaluate the efficacy of the TDA pipeline with a real clinical dataset. Detailed descriptions of these pipelines and statistical methods can be found in Section III and Section IV.

Our key contributions include:

1. **Novel TDA pipeline for preserving temporal dynamics**: We introduce a novel topological data analysis (TDA) pipeline specifically designed for processing resting state fMRI datasets that preserves the temporal dynamics of functional connectivity networks, enabling more accurate and robust analysis of brain connectivity patterns.
2. **Evaluate pipeline accuracy**: We evaluate the performance of our TDA pipeline on a resting state fMRI dataset comprising 371,616 adjacency matrices from 316 subjects at three temporal frequencies. To ensure the efficacy of the TDA-based pipeline, we compare it with three alternative approaches: direct time-series clustering, PCA-based dimensionality reduction and clustering, and traditional fully connected network analysis pipeline with MDS-based dimensionality reduction technique.
3. **Robustness to non-neural variability**: Additionally, we investigate the robustness of the TDA pipeline to variations in data acquisition parameters, specifically examining the impact of temporal sampling rates mitigating the influence of non-neural variability on temporal fMRI datasets.
4. **Validation on multi-site ADHD dataset**: We further validate our method on the ADHD-200 dataset to test generalizability in a multi-site setting with heterogeneous scanning protocols. The TDA pipeline achieves higher inter-site consistency in cluster structure and better subject-level reproducibility, confirming its applicability to broader clinical and developmental neuroscience settings.
5. **Open-source implementation for reproducibility**: We open-source the source code, documentation, and data for all components of our work on GitHub (https://github.com/harp-lab/TemporalBrainPH/), ensuring reproducibility and accessibility.

The remainder of this paper proceeds as follows. First, Section II provides background on related work and the progression of topology-based functional connectivity network analysis. Next, Section III introduces our proposed end-to-end topological data analysis pipeline for temporal brain rs-fMRI data, and Section IV describes the nonTDA-based data processing pipelines for comparison. Section V then presents an evaluation of our TDA pipeline by applying it to analyze temporal dynamics in resting-state fMRI datasets. Finally, Section VI offers a discussion of the results and implications, and Section VII concludes with a summary of our contributions and directions for future work.

## II. RELATED WORK

The application of topological data analysis (TDA) in network analysis extends beyond conventional graph theory, harnessing computational topology tools to characterize network or data structure architectures with greater adaptability [20]. TDA-driven technique has also demonstrated encouraging outcomes in modeling transitions among brain states within fMRI datasets [21]. Persistent homology, as an advanced tool of TDA, is being applied to analyze the topological features of data, providing a powerful framework for studying the evolution and persistence of structural patterns across various scales [22], [23], [24], [25]. The use of persistent homology to analyze functional connectivity networks (FCNs) from resting state fMRI data is on the rise [12], [13], [14], [26]. Persistent homology can quantify structural changes in time-varying graphs, providing both topological summaries and visualizations to identify temporal patterns and anomalies [27]. Recently, we demonstrated that FCN metrics are statistically similar across varied sampling periods [8] only for TDA and not for traditional pipelines. This suggests persistent homology provides a robust topological representation of FCNs invariant to acquisition parameters, potentially removing noise in multi-site studies and improving group comparison effect sizes. Multi-site fMRI studies, despite their advantages in increasing sample size and generalizability [28], [29], introduce variability across scanners and protocols that can undermine statistical power and validity. Differences in acquisition parameters and processing methods across sites can lead to non-biological variability in functional connectivity metrics, posing challenges in large-scale fMRI research [30]. Furthermore, multi-site designs can potentially impact the measurement of temporal dynamics in fMRI studies.

The study of temporal variability in rs-fMRI data has gained significant attention in recent years, as it provides valuable insights into the dynamic nature of functional brain networks and their relevance to human cognition [31]. Growing evidence suggests that functional brain networks exhibit temporal variability, reflecting the dynamic interplay between different cognitive states, arousal levels, and external stimuli [32]. One of the widely used techniques for characterizing temporal variability in rs-fMRI data is the sliding-window correlation analysis. This approach involves dividing the time series into overlapping temporal windows and estimating FCNs for each window [32], [33]. By tracking the changes in functional connectivity patterns across these windows, the dynamic nature of brain networks can be captured, and their evolution over time can be investigated. However, this method relies on predefined window lengths and may not fully capture the inherent temporal dynamics of the data. To address these challenges, existing techniques for analyzing FCNs derived from rs-fMRI have largely relied on graph theory to compute metrics like clustering coefficient and node degree [34], [35]. These measures summarize individual weighted networks and enable comparisons between FCNs, either at the level of nodes/links or using whole-network summaries. However, graph-based methods have significant limitations. First, common graph metrics depend heavily on the parcellation scheme used to define network nodes [36]. Second, graph analysis requires binarizing network links based on an arbitrary threshold, discarding valuable weighting information [37]. While weighted graph analysis has been proposed, such measures still vary with network density [10]. Multi-threshold analysis can mitigate this issue but remains constrained by density effects [38].

Principal component analysis (PCA) is another a useful technique for reducing the high dimensionality of rs-fMRI datasets before applying clustering [39]. By transforming the data into a lower dimensional space of uncorrelated principal components, PCA enables more efficient and robust clustering of large-scale rs-fMRI data [40]. Studies have shown PCA preprocessing can remove noise and highlight the most informative spatial and temporal features in rs-fMRI for improved clustering performance [41], [42] compared to direct clustering approaches [43]. Another approach to characterizing temporal variability is through temporal independent component analysis (ICA) [44]. Temporal ICA aims to identify temporally distinct functional modes or networks from the rs-fMRI data, revealing the underlying temporal structure and dynamics of brain activity. The applicability of temporal principal component analysis (PCA) as a preprocessing step for sliding-window spatial independent component analysis (sICA) of rs-fMRI data is evaluated by analyzing the consistency of PCA-retained subspaces across overlapping time windows [45]. While PCA-based dimensionality reduction has proven useful for rs-fMRI clustering, it is limited to capturing linear relationships and may miss critical topological features in complex functional connectivity networks. Advanced network science techniques are needed to overcome these limitations and characterize topology more comprehensively in a manner invariant to connectivity density or regional parcellation. For example, topological data analysis (TDA) provides a powerful framework for analyzing weighted networks across scales using methods like persistent homology. TDA yields topological summaries that are stable to network density variation. Further, TDA can be applied in a resolution-invariant manner using expansive parcellations. By moving beyond graph theory, TDA-based network analysis can uncover robust and informative topological signatures while avoiding common density and parcellation dependencies. More recently, TDA techniques, such as Mapper and persistent homology, have been introduced as promising tools for studying the temporal variability of rs-fMRI data. Mapper offers a low-dimensional representation of the high-dimensional rs-fMRI data while preserving its topological features [21]. This technique can be particularly useful for visualizing and exploring the temporal variability and overall structure of functional connectivity patterns. Persistent homology, on the other hand, captures the evolution and persistence of topological features (e.g., connected components, loops, voids) across different scales or time points, providing a quantitative measure of the stability and dynamics of functional brain networks [8]. Recently, Chung et al. demonstrated the use of a TDA technique that estimates the state spaces of dynamically changing functional brain networks during resting state by clustering based on the Wasserstein distance metric [46] and proposed a dynamic-TDA framework to distinguish topological patterns of gender-specific brain networks [47]. Furthermore, TDA is well-suited for characterizing changes in network topology over time, providing new avenues for investigating temporal dynamics in fMRI connectivity [48], [49].

## III. TOPOLOGICAL DATA ANALYSIS BASED TEMPORAL CLUSTERING PIPELINE

In this section, we present our end-to-end topological data analysis-based clustering pipeline targeted for temporal resting state fMRI datasets that preserve the temporal dynamics of the high dimensional brain networks. This pipeline masks out non-neural variability in temporal fMRI datasets and demonstrates similarity across temporal cohorts acquired at different sampling frequencies. Our TDA pipeline integrally relies on persistent homology, which, unlike traditional graph analytic approaches, permits analyzing a *range of thresholds* to gather connectivity information from FCNs.

**FIGURE 1.**
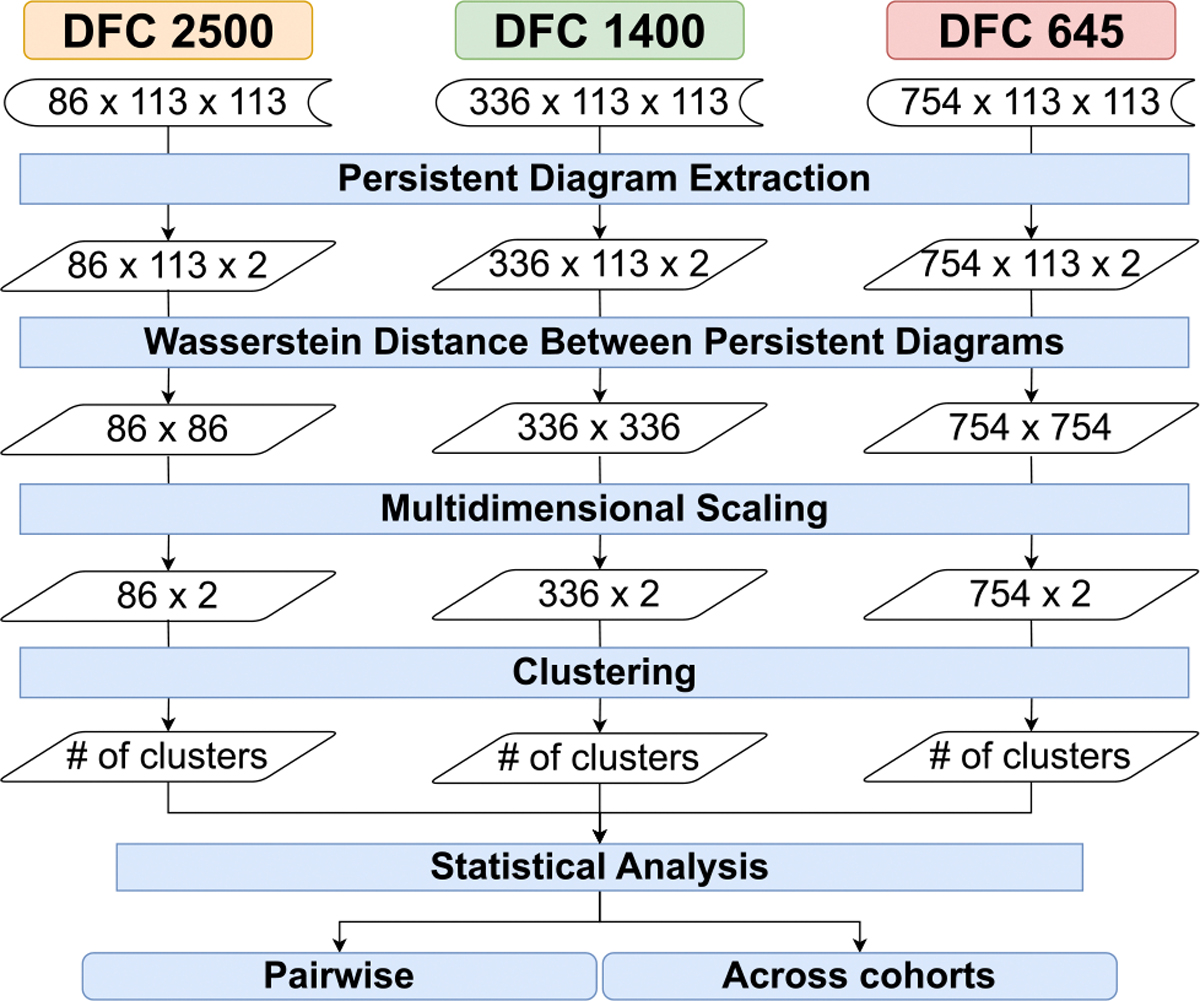
Topological data analysis based temporal clustering pipeline for evaluating the robustness of persistent homology applied to dynamic functional connectivity (DFC) matrices calculated from rs-fMRI data acquired using three different sampling periods for the same subjects ($f_{\mathrm{low}}\;=\;2500\mathrm{ms}$(left), $f_{\mathrm{medium}}\;=\;1400\mathrm{ms}$(center), and $f_{\mathrm{high}}\;=\;645\mathrm{ms}$(right)).

Our processing pipeline aims to exploit the commonly known fact that the resting-state brain typically oscillates between a handful of discrete states [50], [51]. This implies that it is potentially possible to group the temporal timesteps into a discrete number of states. This inherently is a data clustering problem. Therefore, at the heart of our data processing pipeline, we have data clustering across time, which is applied to topological features extracted from the rs-fMRI datasets. A schematic representation of our data processing pipeline can be seen in figure 1. Here we can see the inputs are the three temporal cohorts acquired from sampling 316 subjects at three temporal frequencies ($f_{\mathrm{low}}=2500ms,\;f_{\mathrm{medium}}=1400ms$, and $f_{\mathrm{high}}=645ms$).

### A. MATHEMATICAL FOUNDATIONS AND APPLICATION OF PERSISTENT HOMOLOGY TO RESTING-STATE FMRI

Persistent homology (PH) is an algebraic topology method from Topological Data Analysis (TDA) domain used to study qualitative features of data across multiple scales. It operates on the principle of building simplicial complexes, which are geometric constructs consisting of vertices, edges, triangles, and their higher-dimensional counterparts. These complexes evolve over a range of parameters, providing insights into the topology of the underlying dataset.

Formally, for a given set of points (point cloud) X, persistent homology tracks the birth and death of topological features as a parameter ϵ varies. At each threshold ϵ, a Vietoris-Rips complex ${ℛ}_{\epsilon }(X)$ is constructed by connecting points $x_{i},x_{j}\in X$ if the distance between them $d\left(x_{i},x_{j}\right)$ is less than or equal to ϵ. As ϵ grows, simplices (vertices, edges, triangles) appear and merge, reflecting changes in topology. Persistent homology captures these changes as persistent barcodes or persistence diagrams, encoding the lifespan (birth to death intervals) of topological features.

In the context of rs-fMRI, PH provides a robust methodology to analyze FCNs, which represent interactions between brain regions as correlation-based adjacency matrices. Direct analysis of these networks can be challenging due to high dimensionality and inherent noise. Persistent homology addresses these challenges by interpreting each brain region as a vertex, forming Vietoris-Rips complexes based on distances derived from correlations. By varying the threshold ϵ, persistent homology captures and quantifies the emergence and merging of connected components, offering robust summaries of complex temporal dynamics.

Our pipeline specifically employs 0-dimensional persistent homology to characterize the evolution of connected components within dynamic FCNs. This approach facilitates robust and interpretable topological signatures suitable for temporal clustering analyses across diverse data acquisition parameters and sampling frequencies.

Our end-to-end TDA pipeline has the following steps:

1. **Generate FCNs from rs-fMRI data**: We applied dynamic-windowed Pearson correlation on the fMRI dataset (as used by us before in Jia et al [4]) to generate the dFCNs from data acquired with different acquisition parameters ($f_{\mathrm{high}}=645ms,\;f_{\mathrm{medium}}=1400ms$, and $f_{\mathrm{low}}=2500ms$.) as a data pre-processing step (Section III-B). This provides an FCN at each time point.
2. **Create distance matrix from FCNs**: The extracted FCNs at each time point are then converted into distance matrices as a weighted graph (Section III-C).
3. **Extract persistent barcodes from distance matrix**: In this step, we extract persistent diagrams (0-dimensional barcodes) from the distance matrices using persistent homology to identify the topological features from the matrices (Section III-D).
4. **Temporal clustering on PD to prove the resiliency of different data acquisition parameters**: Finally, we apply temporal clustering on the extracted barcodes to see the similarity between the topological features extracted using different data acquisition parameters (HYPERLINK Section III-E).

### B. FCN GENERATION FROM RS-FMRI DATA

We sourced the structural T1-weighted and rs-fMRI data from the freely accessible Enhanced Nathan Kline Institute Rockland Sample database (NKI-RS) [52]. The MRI data was collected using a 3T Siemens Magnetom Tim Trio scanner. The acquisition parameters for the T1-weighted structural data included: isotropic voxels of 1.0 mm with 176 slices, a repetition time (TR) of 1900 ms, an echo time (TE) of 2.52 ms, and a field of view (FOV) of 250 × 250. Resting state fMRI data was gathered using multiband echo-planar imaging (EPI) [53] from each participant using three distinct acquisition protocols with varying parameters. The first protocol used 3.0 mm isotropic voxels with 40 slices, a TR of 645 ms, a TE of 30 ms, a FOV of 222 × 222 mm, 900 volumes, and a multi-band factor of 4. The second protocol used 2.0 mm isotropic voxels with 64 slices, a TR of 1400 ms, a TE of 30 ms, a FOV of 224 × 224 mm, 404 volumes, and a multi-band factor of 4. The third protocol used 2.0 mm isotropic voxels with 38 slices, a TR of 2500 ms, a TE of 30 ms, a FOV of 216 × 216 mm, 120 volumes, and a multi-band factor of 1. Even though the three datasets from each participant are identified with the corresponding TR, they differ in several other scan parameters, such as the number of volumes, multi-band factor, FOV, and voxel size. The MRI data underwent a standard pre-processing pipeline, which included the removal of the first five volumes, slice time correction, and motion correction. T1-weighted anatomical images were aligned to the mean functional images, which were then spatially registered to a standard MNI152 template. Nuisance variables such as low-frequency drifts, and motion parameters were regressed out. Unwanted physiological fluctuations (signals from white matter and cerebrospinal fluid) were eliminated using aCOMPCor (anatomical component-based noise correction). After excluding subjects that did not pass quality control, we identified 316 subjects with usable data from all three acquisition protocols. We then obtained mean time series from 113 brain regions (using the Yeo parcellation template [54]) for each subject and acquisition protocol. Using Pearson’s correlation, we estimated FCN matrices from these mean time series.

**FIGURE 2.**
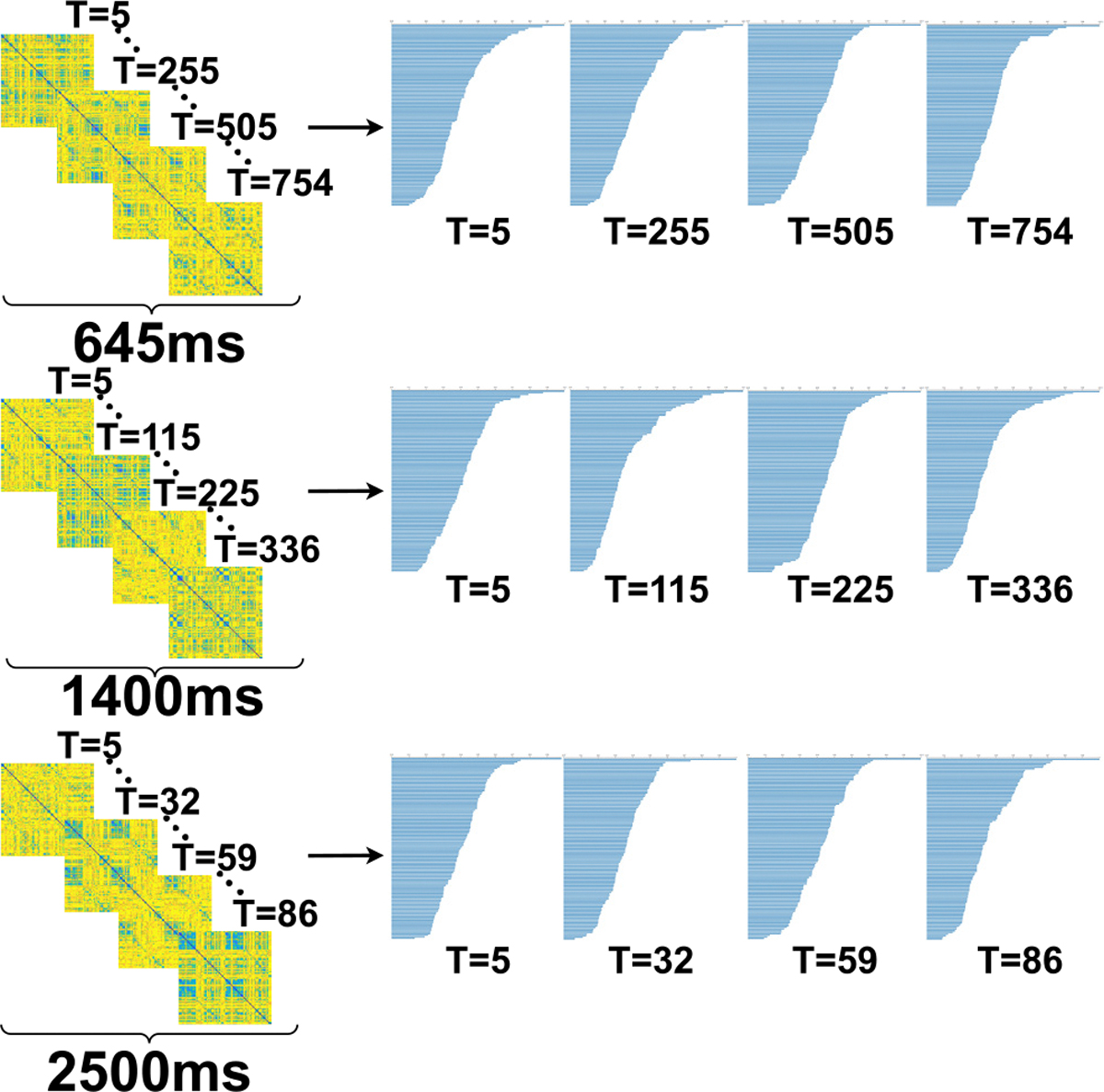
The FCN and extracted topological feature as 0-dimensional barcodes for subject 32 at different time points for temporal sampling periods, i.e. high temporal frequency ($f_{\mathrm{high}}\;=\;645\mathrm{ms}$) (top), medium temporal frequency ($f_{\mathrm{medium}}\;=\;1400\mathrm{ms}$) (center), and low temporal frequency $f_{\mathrm{low}}\;=\;2500\mathrm{ms}$ (bottom). The matrix size is 113 × 113, corresponding to 113 brain regions.

At this stage, we generate one FCN for each timepoint using dynamic-windowed Pearson’s correlation as in our previous work [4]. Briefly, this method uses sliding temporal windows to calculate Pearson’s correlation to be assigned to each time point. The width of the temporal window is dynamically determined by the stationarity of the statistical properties of the time series within the window, determined by the Augmented Dickey-Fuller test. Each FCN is stored as a symmetric adjacency matrix M with size 113 × 113, where $M_{ij}$ represents the correlation coefficient between brain nodes i and j. The dataset consists of three temporal frequencies ($f_{\mathrm{high}}=645ms$,$f_{\mathrm{medium}}=1400ms$, and $f_{\mathrm{low}}=2500ms$). There are 316 subjects for each temporal frequency. High temporal frequency ($f_{\mathrm{high}}=645ms$) yields a total of 754 time-steps; medium temporal frequency ($f_{\mathrm{medium}}=1400ms$) scan yields 336 time-steps; and on the other end of the spectrum is low-frequency scans of $f_{\mathrm{low}}=2500ms$ yields 86 time-steps. The total number of adjacency matrices is 371,616(316 × 754) + (316 × 336) + (316 × 86) with a dimension of 113 × 113. Figure 2 (left column) shows an example of the extracted FCNs for subject 32, for the three sampling periods at different time points.

### C. CREATION OF MATRICES FROM FCNS

Usually, topological data analysis uses point cloud data in metric configuration. We confine the weighted networks from fMRI data in distance matrices in our TDA pipeline before applying TDA techniques. Then, we extract persistent barcodes from the distance matrices.

We use popular Pearson’s correlation coefficients $pcc\left(p_{t},q_{t}\right)$ to measure the linear correlation between any two data points (nodes) $p_{t}$ and $q_{t}$ at time t in the fMRI data [55]. However, correlation coefficients do not directly represent distances, which are often required for clustering algorithms or other analyses. Therefore, we transform the correlation coefficients into a distance metric using the following formula:

$$d\left(p_{t},q_{t}\right)=\sqrt{1-pcc{\left(p_{t},q_{t}\right)}^{2}}$$

This transformation ensures that higher positive correlations (closer to 1) are mapped to smaller distances (closer to 0), while lower correlations (closer to 0) or negative correlations are mapped to larger distances. The temporal indexing t indicates this is a temporally dynamic dataset, and the correlations and distances are calculated between node pairs at each time point.

### D. EXTRACTION OF PERSISTENT BARCODES FROM DISTANCE MATRICES

**FIGURE 3.**
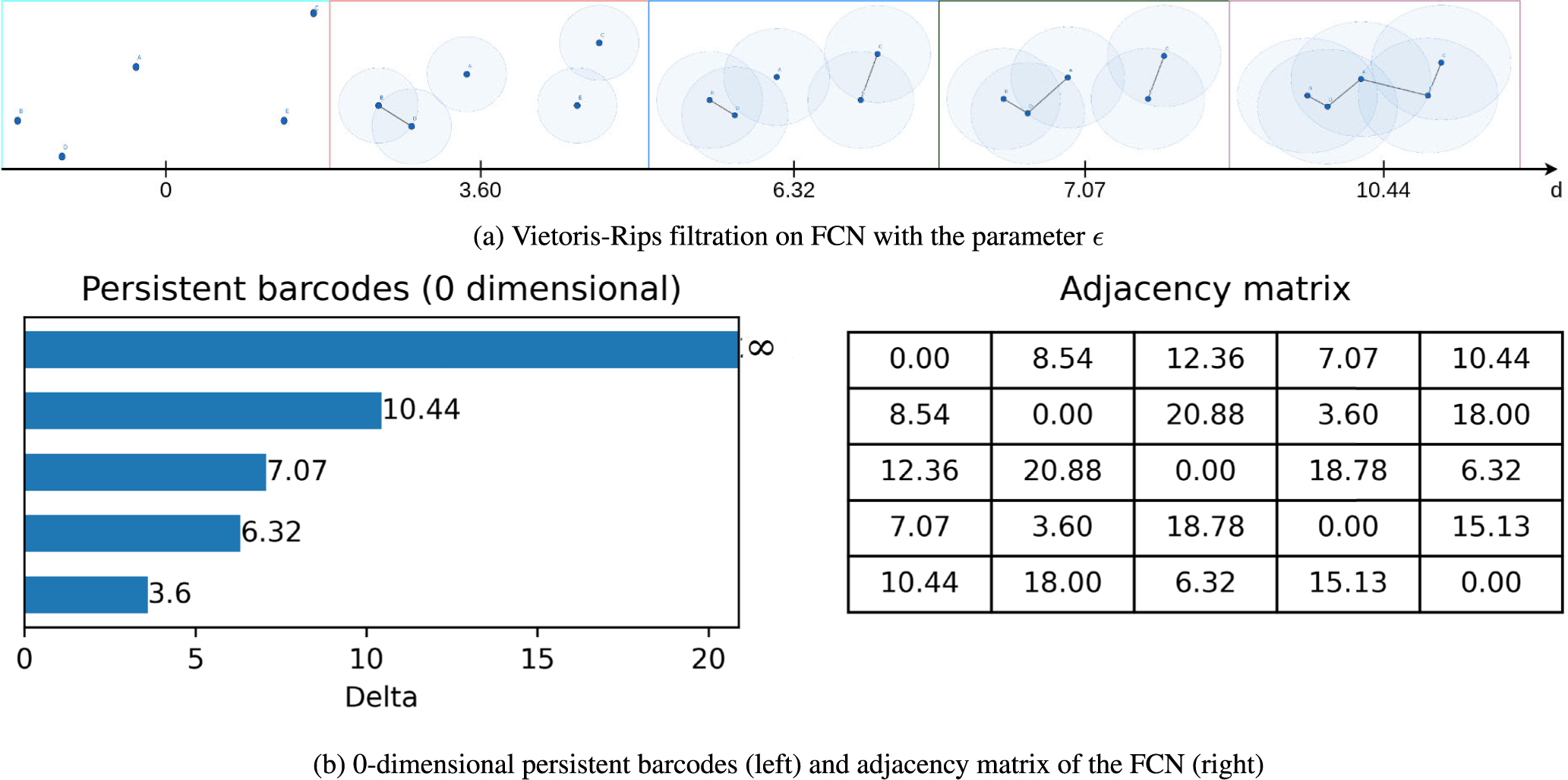
An example of persistent homology to extract topological features using 0-dimensional barcodes. The adjacency matrix is of size 5 × 5.

Unlike traditional graph analytic approaches, persistent homology permits analyzing a range of thresholds to gather connectivity information from a given FCN. Figure 3 shows an example of using persistent homology to record the changes of topological features over the changes of distances using zero-dimensional barcodes. Vietoris-Rips filtration on the given 5 × 5 FCN is applied to capture the changes in the number of connected components for different parameters of d. Using persistent homology, we capture topological features from the distance matrices extracted from the fMRI FCNs. This section overviews topological feature extraction using persistent barcodes and the distance metric we have used. The existing literature contains the details on these topics [24], [56].

#### 1) EXTRACTION OF TOPOLOGICAL FEATURES USING PERSISTENT HOMOLOGY

Persistent homology can extract topological features from a topological space. The homology of the space can be divided into groups based on the dimensions of the features. A topological space X can be divided into homology groups $H_{i}(X)$ for i=0,1,2,… where $H_{i}(X)$ represents the $i^{t}h$ homology group. Each homology group $H_{n}(X)$ denotes the number of n-dimensional holes in the topological space X. For example, the $H_{0}(X)$ homology group shows the number of connected components, $H_{1}(X)$ homology group shows the number of holes, $H_{2}(X)$ homology group shows the number of voids in the topological space X.

In this paper, we use $H_{0}(X)$ homology group (0-dimensional) to extract the number of connected components from the rs-fMRI FCNs (at each time point) as topological features. Figure 3 shows a simple example of using persistent homology to extract the topological features from a given FCN. The table at the bottom right in Figure 3(b) represents the adjacency matrix of an FCN with five nodes. Persistent homology captures the changes in topological features over distance thresholds (ϵ) between the nodes. In a point cloud, F with p nodes, two nodes (x,y) are marked as connected with an edge if the distance d(x,y) is less than threshold ϵ. In this scenario, they form a 1-Simplex. When three nodes are connected with each other for some value of ϵ, they form 2-Simplex and so on. For a given ϵ, the graph is called a Rips complex represented by Rips(F,ϵ). These continuous changes in the value of ϵ result in the changes in topological features. Vietoris-Rips filtration captures the increasing value of ϵ for which a new Rips-complex, in other words, a new topological feature, is being generated [57]. Figure 3(a) shows the extraction of different topological features in various thresholds of ϵ using Vietoris-Rips filtration.

In this example, for each real number t where topological features are changed, we consider them important events and store these values of t. Here, t represents the filtration value used in the persistent homology analysis, which is derived from the adjacency matrix (bottom right table of Figure 3) representing the FCN. As the filtration value t increases, topological features (such as connected components) can appear, merge, or disappear in the simplicial complex constructed from the data. For instance, at some time $t_{0}$, a topological feature, a component is being created, and at time $t_{1}$, it is merged with another component. We keep track of the birth as $t_{\mathrm{birth}}=t_{0}$ and death as $t_{\mathrm{death}}=t_{1}$ for each component. The time of the ($\left.t_{\mathrm{birth}},t_{\mathrm{death}}\right)$ of the topological features is visualized as *barcodes*. The span of time for each feature $t_{\mathrm{death}}-t_{\mathrm{birth}}$ is called the persistence of that feature. Figure 3(b)(left) shows the barcodes for the given FCN. At t=0, five topological features are born as five independent (connected) components. At t=3.6, two components are merged; thus, the death of a component is recorded at t=3.6. Therefore, the persistence of that component is 3.6. Similarly, at t=6.32, another component is merged with a persistence of 6.32. For 0-dimensional persistence barcodes, this process continues until there is only one connected component. This last component never dies; thus, the persistence of this component is ∞. The 0-dimensional persistence barcodes in Figure 3(b)(left) represent the *birth* and *death* of the topological features, which is the changes in the number of connected components of the FCN. Each horizontal bar begins at the birth of a component and ends at the death of each component in the barcodes representation. While higher-order features such as 1-dimensional homology (loops) can in principle be extracted, we restrict our analysis to $H_{0}$ for two reasons: (i) computational tractability on large, time-varying FCNs, and (ii) prior evidence that connected components provide stable and interpretable topological features for functional brain networks.

#### 2) FORMATION OF PERSISTENT DIAGRAMS AS SIGNATURES FOR THE FCNS

The 0-dimensional barcodes extracted from the functional connectivity networks (FCNs) represent the evolution of connected components over distance thresholds. In our pipeline, we generate 0-dimensional persistent barcodes for each FCN. Each FCN has 113 vertices that form the finite set of points F where the value $d_{i}$ represents the pairwise distance between the points. We extract 0-dimensional barcodes using persistent homology for all the 371,616 FCNs ((316 × 86) + (316 × 336) + (316 × 754)). Figure 2 displays illustrations of the extracted barcodes at various time points across three different temporal sampling periods ($f_{\mathrm{high}}=645ms,\;f_{\mathrm{medium}}=1400ms$, and $f_{\mathrm{low}}=2500ms$) for a single subject (subject 32). After this stage, we get timestep# × 113 × 2 as 0-dimensional barcodes for each of the sampling periods.

A persistent barcode can be represented with a persistent diagram without information loss where the birth and death of a component (a topological feature) are represented as a point on the X-axis and Y-axis, respectively. These points on a two-dimensional surface as a *persistent diagram* can be used for statistical inference to prove that persistent homology is resilient to different data acquisition parameters. By forming the persistent diagrams from the 0-dimensional barcodes of each FCN, we obtain topological signatures that quantify the evolution of connected components over distance thresholds in the functional brain networks. The distance between points in the persistent diagram provides a stability measure, allowing us to compare topological signatures across subjects. Thus, these signatures are used as features for statistical analysis in our experiments.

### E. TEMPORAL CLUSTERING ANALYSIS

The literature shows the usability of earth moving distance, also known as Wasserstein distance (WD), for statistical inference of persistent diagrams [15], [16]. We use WD as a metric to compute the distance between two persistence diagrams extracted from two FCNs. WD represents the minimum value that is computed in the match calculation between the points of two persistent diagrams. The WD value of two similar persistent diagrams is smaller than two dissimilar persistent diagrams. This WD metric assists in proving the hypothesis of similarity between the persistent diagrams extracted from different data acquisition parameters, such as sampling rates.

The temporal clustering analysis is to evaluate our hypothesis of the similarity of the fMRI FCNs obtained from different data acquisition parameters, such as different temporal sampling rates (TR). Our dataset includes three data cohorts: $f_{\mathrm{high}}=645ms,\;f_{\mathrm{medium}}=1400ms$, and $f_{\mathrm{low}}=2500ms$. High temporal frequency ($f_{\mathrm{high}}=645ms$) has a total of 754 time-steps; medium temporal frequency ($f_{\mathrm{medium}}=1400ms$) scan yields 336 time-steps; and low temporal frequency $f_{\mathrm{low}}=2500ms$ yields 86 time-steps for each of the subjects. Figure 1 represents the TDA pipeline we develop for the TDA framework. We calculate pairwise WD between the persistent diagrams of the timesteps for all subjects within the same data cohort.

Let,

$$S_{i}=\;\mathrm{Subject}\;i,\;\mathrm{where}\;i\in \{1,\;2,\ldots ,316\}$$

$$T_{k}=\;\mathrm{Number}\;\mathrm{of}\;\mathrm{time}\;\mathrm{steps}\;\mathrm{for}\;\mathrm{data}\;\mathrm{cohort}\;k,$$

where $k\in \left\{754,\;336,\;86\right\}$

$$P_{S_{i},t}=Persistence\;diagram\;for\;subject\;S_{i}\;at\;time\;t,$$

where $t\in \left\{1,\;2,\ldots ,T_{k}\right\}$

$$d_{W}\left(P_{S_{i},t},P_{S_{j},t}\right)=Wasserstein\;distance\;between\;P_{S_{i},t}\;and\;P_{S_{j},t}$$

The TDA distance matrix for a given subject $S_{i}$ in cohort k is defined as:

(1) $$D{\left(S_{i}\right)}_{t_{1},t_{2}}=d_{W}\left(P_{S_{i},t_{1}},P_{S_{i},t_{2}}\right)\;\;\mathrm{for}\;t_{1}\in \left\{1,\;2,\ldots ,T_{k}\right\}\times t_{2}\in \left\{1,\;2,\ldots ,T_{k}\right\}$$

In Eq. 1, we compute the pairwise Wasserstein distance between persistence diagrams at times $t_{1}$ and $t_{2}$, which gives a $T_{k}\times T_{k}$ distance matrix for each subject $S_{i}$. We have three data cohorts ($f_{\mathrm{high}}=645ms,\;f_{\mathrm{medium}}=1400ms$, and $f_{\mathrm{low}}=2500ms$) with different timesteps (754, 336, 86). Thus, we get 316 adjacency matrices for each data cohort with size (754 × 754), (336 × 336), and (86 × 86), respectively.

These high-dimensional adjacency matrices are complex and cannot be analyzed by statistical methods. To make it interpretable by the statistical methods and for better visualization, we apply the multidimensional scaling (MDS) technique to reduce the dimensionality of the matrices. After this stage, we get 316 adjacency matrices for each data cohort with sizes (754 × 2), (336 × 2), and (86 × 2).The two-dimensional MDS results are plotted using scatter plots that give an intuition to use clustering to calculate the similarity between the TRs. We apply the k-means clustering technique to the MDS results to get the number of clusters for the reduced-sized matrices. As k-means clustering requires configuring the number of clusters n before the cluster computation, we choose n using a well-known approach called Silhouette analysis. Using Silhouette analysis, we choose n that gives the maximum Silhouette score for the given adjacency matrices between the range from 2 to 16 [58], [59]. After calculating the number of clusters for all three data cohorts, we get the number of clusters for all 316 subjects across these data cohorts. A similar number of clusters for the same subject across three data cohorts will indicate the similarity between the subjects for different data acquisition parameters (different TRs in our case). If we get significant similarity between the TRs, we can conclude the resiliency of persistent homology for dynamic functional connectivity derived from rs-fMRI with different data acquisition parameters.

We use Matlab during the data preprocessing steps and Python for statistical analysis. For the topological data analysis framework, we use *Gudhi* library [60], [61] to compute the 0-dimensional barcodes from FCNs and to calculate WD distances between the persistent diagrams. We also use *scikit-learn* library for multidimensional scaling and cluster calculation [58].

## IV. NONTDA-BASED DATA PROCESSING PIPELINES FOR RS-FMRI DATASET

To assess the effectiveness of our TDA pipeline presented in Section III, we implement three alternative nonTDA-based data processing pipelines for comparative analysis. Section IV-A provides a detailed walkthrough of a direct time-series clustering pipeline. In Section IV-B, we show a pipeline that employs Principal Component Analysis (PCA) for dimensionality reduction and clustering. Section IV-C outlines a traditional functional connectivity network (FCN) analysis pipeline tailored for brain network datasets. As the datasets are in *mat* format, for data preprocessing, we utilize Matlab to process the raw fMRI time series, handle missing values, and construct functional connectivity networks. Python is used for all subsequent analyses, including dimensionality reduction, clustering, and statistical testing. We use *scikit-learn* library for principal component analysis, multidimensional scaling, and k-means clustering [58].

### A. DIRECT TIME-SERIES CLUSTERING PIPELINE

**FIGURE 4.**
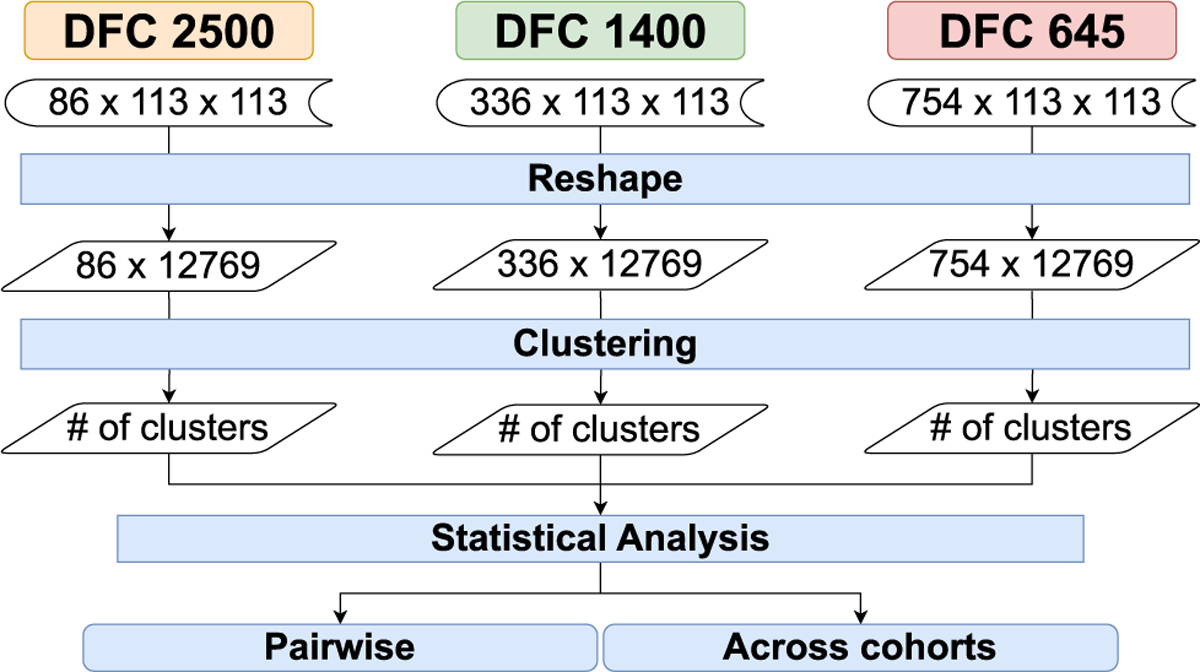
Direct time-series clustering pipeline that bypasses dimensionality reduction of the high dimensional temporal datasets.

Our first baseline pipeline is the nonTDA-based direct clustering pipeline. It implements a direct clustering approach on the temporal rs-fMRI datasets without any dimensionality reduction or graph construction steps. As shown in Figure 4 the input of this pipeline is the *timestep*# × 113 × 113× where 113×113 corresponds to the spatial resolution of each of the individual FCN capturing the pairwise connectivity strengths among the 113 spatial regions of the brain. The *timestep*# is respectively 86, 336, and 754 for the three temporal frequencies $f_{\mathrm{low}},\;f_{\mathrm{medium}}$, *and*, $f_{\mathrm{high}}$ respectively.

Directly clustering multivariate FCNs poses challenges due to the high dimensionality of the data. To mitigate this, we flatten the 113 × 113 matrices into one-dimensional arrays of length 12, 769, resulting in *timestep*# × 12, 769 sized matrices for each of the subjects for each data cohort prior to clustering. This reshaping transforms the data into a format amenable to traditional clustering algorithms. Similar to the TDA pipeline, we then apply the k-means clustering technique to reshaped matrices. We utilize the silhouette analysis method to determine the optimal number of clusters, denoted as n, within the range of 2 to 16, ensuring the selection of the cluster configuration with the highest silhouette score.

After calculating the number of clusters for all three data cohorts, we get the number of clusters for all 316 subjects across these data cohorts. The subsequent step defines the statistical analysis phase, where we perform pairwise and cohort-wide set overlaps of the number of clusters. Similar to the TDA pipeline, the key hypothesis is that robust clustering solutions should exhibit consistency across subjects and sampling rates. To evaluate this, we statistically compare the identified cluster numbers across cohorts using pairwise and group-wise similarity scores. Higher overlaps in the optimal cluster numbers between the three temporal sampling periods and higher pairwise set overlaps will indicate higher similarity scores, thereby affirming the robustness of the direct clustering approach to variability in sampling rates.

### B. PCA-BASED DIMENSIONALITY REDUCTION AND CLUSTERING PIPELINE

**FIGURE 5.**
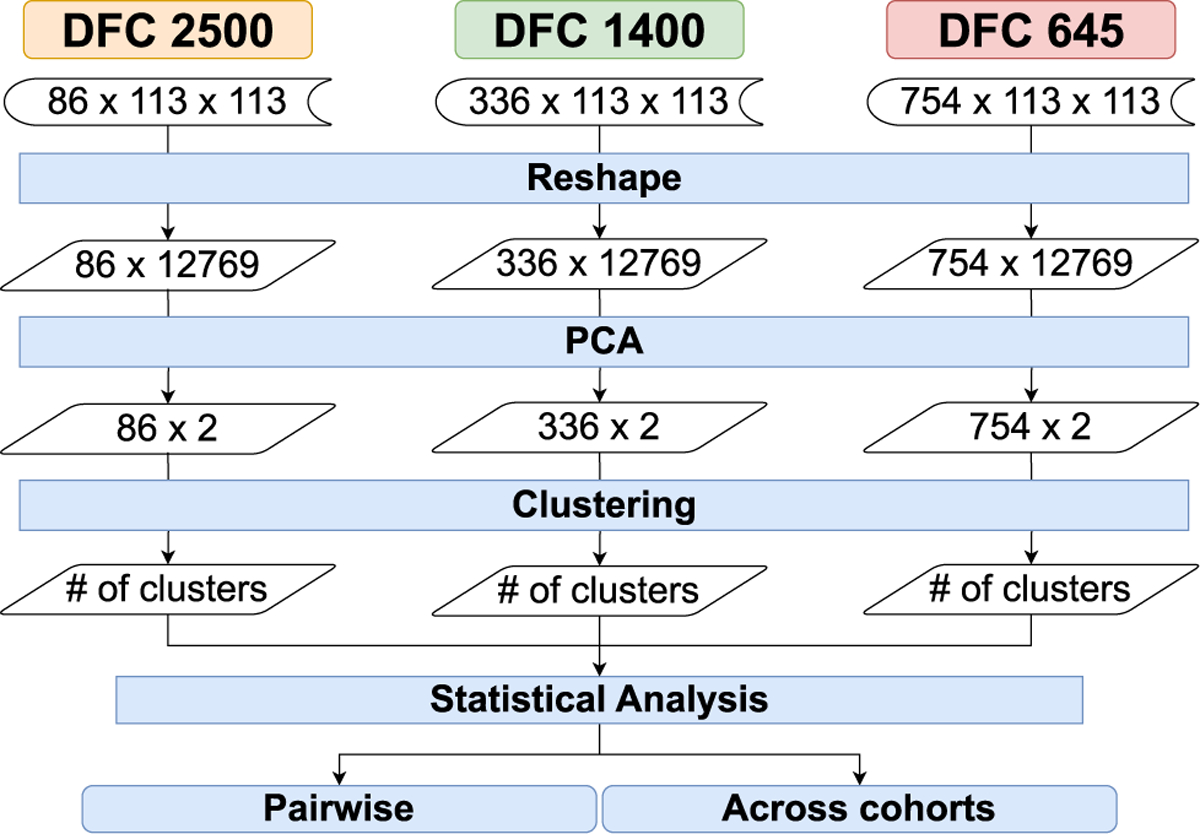
PCA-based dimensionality reduction and clustering pipeline where the high dimensional data is reduced to 2 principal components capturing the most variance before applying clustering algorithm.

After establishing the baseline with the direct clustering approach, we introduce a principal component analysis (PCA)-based dimensionality reduction and clustering pipeline to address the high dimensionality challenges in the temporal FCN datasets. Figure 5 displays PCA-based dimensionality reduction and clustering pipeline. This pipeline incorporates a three-step process involving PCA for dimensionality reduction, k-means clustering, followed by statistical analysis.

Like the prior pipelines, this pipelines takes as input matrices of size *timestep*# × 113 × 113. where the *timestep*# values are 86, 336, and 754 for the temporal frequencies $f_{\mathrm{low}},\;f_{\mathrm{medium}}$, and, $f_{\mathrm{high}}$, respectively. We flatten the 113 × 113 functional connectivity matrices into one-dimensional arrays of length 12, 769 to reorganize our data into a 2D matrix suitable for PCA, where each row represents a timestep and each column represents an element of the flattened connectivity matrix. After flattening, the input to PCA would indeed be a 2D matrix with dimensions: for $f_{\mathrm{low}}:\;86\;\times \;12,\;769$, for $f_{\mathrm{medium}}:336\times 12,\;769$, and for $f_{\mathrm{high}}:754\times 12,\;769.$ This reshaping transforms the higher-dimensional tensor data into a 2D matrix format required by PCA to identify the top principal components capturing the most variance in the connectivity patterns across time. Applying PCA on the flattened 12, 769 dimensional data then allows us to reduce the high dimensionality down to the most informative 2 principal components before clustering. In this stage, we apply PCA to reduce the dimensionality of the flattened *timestep*# × 12, 769 matrices. Specifically, we use PCA to project the data onto a lower-dimensional subspace while retaining as much variance as possible. For each temporal frequency ($f_{\mathrm{low}},\;f_{\mathrm{medium}},\;f_{\mathrm{high}}$), we reduce the dimensions from 86 × 12769 to 86 × 2 for $f_{\mathrm{low}}$, 336 × 12769 to 336 × 2 for $f_{\mathrm{medium}}$, and 754 × 12769 to 754 × 2 for $f_{\mathrm{high}}$. The resulting PCA-transformed matrices, now of size *timestep*# × 2, capture the most salient features of the original data. This dimensionality reduction facilitates subsequent clustering by focusing on the most informative components while significantly reducing computational complexity.

Following the PCA-based dimensionality reduction, we apply k-means clustering to identify patterns and group subjects based on the reduced feature space. Similar to the direct clustering approach, we leverage silhouette analysis to determine the optimal number of clusters (n) within the range of 2 to 16 for each temporal frequency that maximizes clustering quality. The resulting cluster assignments provide a compact representation of the original data while capturing meaningful variations across subjects and temporal sampling rates.

To assess the performance of the PCA-based pipeline, we follow a similar statistical analysis phase as in the direct clustering approach. The number of clusters obtained for all subjects across the three temporal frequencies undergoes pairwise and cohort-wide set overlap analysis. This comparison helps evaluate the consistency of clustering solutions across different temporal sampling periods. The hypothesis remains that a robust clustering solution should exhibit coherence in identified clusters across subjects and temporal frequencies. We use pairwise and group-wise similarity scores to statistically compare the optimal cluster numbers. Higher overlaps in cluster assignments between temporal sampling periods and increased pairwise set overlaps indicate greater stability and reliability in the face of variability in sampling rates.

### C. TRADITIONAL DFCN CLUSTERING PIPELINE WITH MDS-BASED DIMENSIONALITY REDUCTION

**FIGURE 6.**
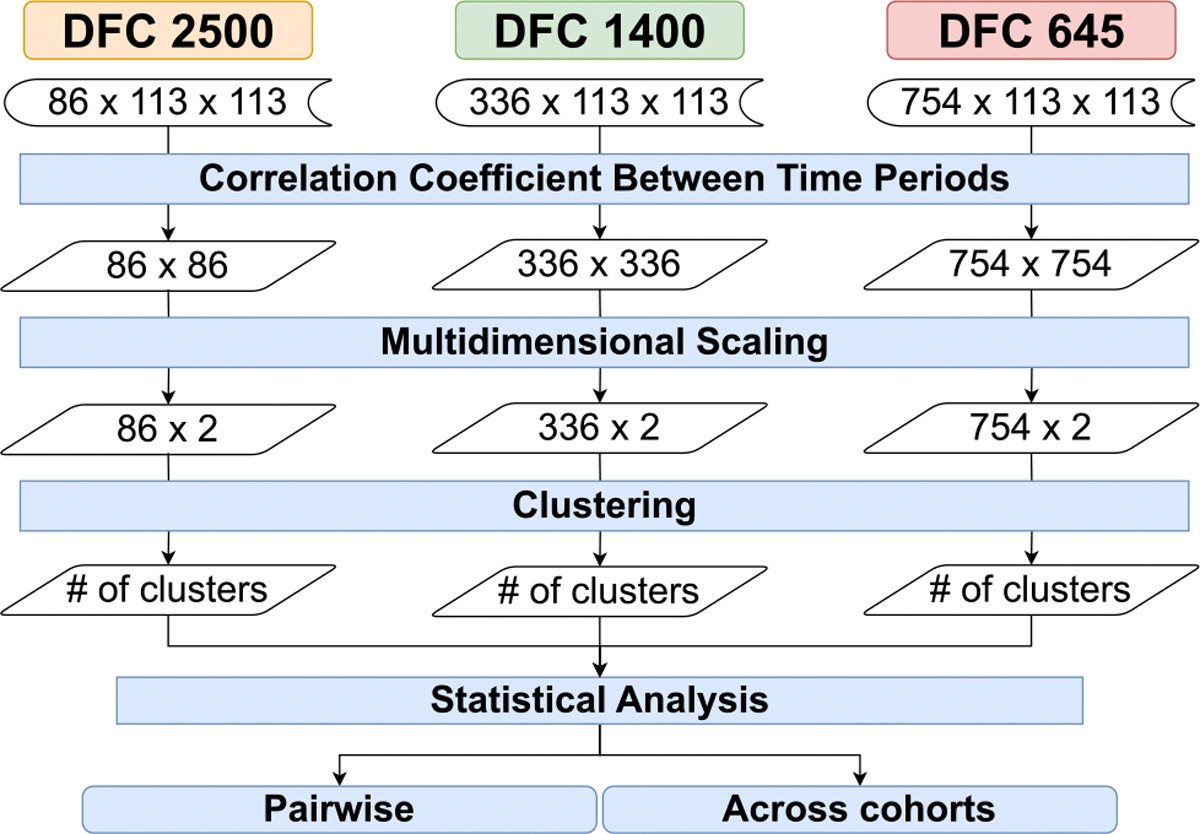
Traditional dynamic functional connectivity network (dFCN) clustering pipeline. This non-TDA pipeline has a similar statistical analysis structure to the TDA pipeline, allowing comparison of approaches for assessing the robustness of persistent homology on temporal rs-fMRI data.

We develop a traditional dynamic FCN (dFCN) analysis pipeline with similar steps to the aforementioned TDA-based pipeline for the rs-fMRI dataset. The traditional dFCN analysis pipeline is illustrated in Figure 6. Analysis steps include extracting subject-specific DFC (dynamic functional connectivity) matrices, calculating graph metrics, dimensionality reduction via MDS, clustering with k-means, and computing cluster overlaps. Instead of the persistent diagrams or applying any persistent homology methods, we use a correlation coefficient between the timesteps for all three data cohorts.

Let,

$$S_{i}=\;\mathrm{Subject}\;i,\;\mathrm{where}\;i\in \{1,\;2,\ldots ,316\}$$

$$T_{k}=\;\mathrm{Number}\;\mathrm{of}\;\mathrm{time}\;\mathrm{steps}\;\mathrm{for}\;\mathrm{data}\;\mathrm{cohort}\;k,$$

where $k\in \left\{f_{\mathrm{high}}=754,f_{\mathrm{medium}}=336,f_{\mathrm{low}}=86\right\}$

$$\begin{array}{l} A_{S_{i},t}=\;\mathrm{Adjacency}\;\mathrm{matrix}\;\mathrm{for}\;\mathrm{subject}\;S_{i}\;\mathrm{at}\;\mathrm{time}\;t,\\ \;\;\mathrm{where}\;t\in \left\{1,2,\ldots ,T_{k}\right\} \end{array}$$

The distance matrix for a given subject $S_{i}$ in cohort k is defined as:

(2) $$D{\left(S_{i}\right)}_{t_{1},t_{2}}=\sqrt{\sum_{m=1}^{M}\;\sum_{n=1}^{N}\;{\left|a_{mn}^{t_{1}}-a_{mn}^{t_{2}}\right|}^{2}}$$

In Eq. 2, M,N are the dimensions of the adjacency matrices. We compute the Euclidean distance [62] between adjacency matrices at times $t_{1}$ and $t_{2}$, which gives 316 distance matrices of sizes (86 × 86), (336 × 336), and (754 × 754) for the three cohorts respectively. In this stage, we acquire 316 matrices for each of the data cohorts with the size of (86 × 86) for $f_{\mathrm{low}}=2500ms$, (336 × 336) for $f_{\mathrm{medium}}=1400ms$, and (754 × 754) for $f_{\mathrm{high}}=645ms$. Then, we follow a similar pipeline of the TDA framework to keep the comparison uniform. We reduce the dimension of the matrices using two-component multidimensional scaling (MDS) and then calculate the number of clusters (n) on the reduced matrices using k-means clustering. We also use the maximum Silhouette score to choose the value of n within the range from 2 to 16. Finally, this pipeline will also produce the number of clusters of the MDS for every 316 subjects for all three data cohorts. Our hypothesis will be proven right if the TDA pipeline gives a better similarity score than the non-TDA pipelines.

## V. RESULTS

All of the TDA-based and nonTDA-based pipelines start with embedding one FCN for each rs-fMRI scan as an adjacency matrix (section III-B). In this stage, we get 371, 616 adjacency matrices ((316 × 86) + (316 × 336) + (316 × 754)) for three temporal sampling periods ($f_{\mathrm{high}}=645ms,\;f_{\mathrm{medium}}=1400ms$, and $f_{\mathrm{low}}=2500ms$). Each matrix has a dimension of 113 × 113. In the second stage of the pipelines, we embed the FCN using Pearson’s correlation coefficients and range the values between 0 and 1. In the TDA-based pipeline, the third stage extracts 0-dimensional persistent barcodes from the matrices using persistent homology (Section III-D). In the nonTDA-based pipelines, instead of using persistent homology, we use correlation coefficients between the timesteps for the three temporal sampling periods (TRs) for traditional FCN analysis(Section IV-C). Both the direct clustering and PCA-based dimensionality and clustering pipeline reshape the input matrices from *timestep*# × 113 × 113 to *timestep*# × 12,769 where the later one applies PCA based dimensionality reduction before applying clustering. For all of the TDA-based and nonTDA-based pipelines, we continue to the statistical analysis phase, where we compute the cohort-wide and pairwise cluster intersections of the subjects for all data cohorts.

In the TDA-based pipeline, we use the Wasserstein distance metric on the persistent diagrams for all the subjects for all three temporal sampling periods. On the contrary, in the nonTDA-based traditional FCN analysis pipeline, we directly use the correlation coefficient on the extracted FCNs. Adjacency matrices generated after this stage in these pipelines are similar in size for respective temporal sampling periods. For temporal sampling period $f_{\mathrm{low}}=2500ms$ with 86 timesteps in the TDA-based pipeline, each subject yields adjacency matrix WD of size (86 × 86) where $WD_{ij}$ represents the pairwise Wasserstein distance between timestep i and j. In the traditional FCN analysis pipeline for the same data cohort, each subject yields adjacency matrix A of size (86 × 86) where $A_{ij}$ represents the pairwise norm between timestep i and j. Similarly, $f_{\mathrm{medium}}=1400ms$ and $f_{\mathrm{high}}=645ms$ yield adjacency matrix of size (336 × 336) and (754 × 754), respectively, for each of the subjects during TDA and traditional FCN analysis. This high dimensionality of the matrix size makes it challenging to apply statistical analysis. For this reason, we applied multidimensional scaling (MDS) and reduced the size of the matrices to fit into a two-dimensional surface for all the data cohorts($f_{\mathrm{low}}:(86\times 2)$, $f_{\mathrm{medium}}:(336\times 2)$, $f_{\mathrm{high}}:(754\times 2)$). Then, we applied clustering on the MDS data using the k-means clustering algorithm with Silhouette analysis to select the number of clusters. Finally, we get the number of clusters for all 316 subjects for both of these pipelines.

For the other two nonTDA-based data processing pipelines, we first reshape the input matrices. In the direct time-series clustering pipeline, we flatten the 113 × 113 matrices into 12, 769-dimensional vectors and calculate the optimal number of clusters for each data cohort directly on this reshaped high-dimensional data. In contrast, for the PCA-based dimensionality reduction and clustering pipeline, we flatten the FCNs and then apply 2-component PCA to reduce the dimensionality down to 2 principal components before clustering. This PCA step mitigates the challenge of directly clustering high-dimensional data. After PCA reduction to 2D, we determine the optimal cluster numbers for each subject on the low-dimensional PCA-reduced data. Both pipelines reshape the data as a preprocessing step, but the PCA pipeline has an additional dimensionality reduction phase prior to clustering.

**FIGURE 7.**
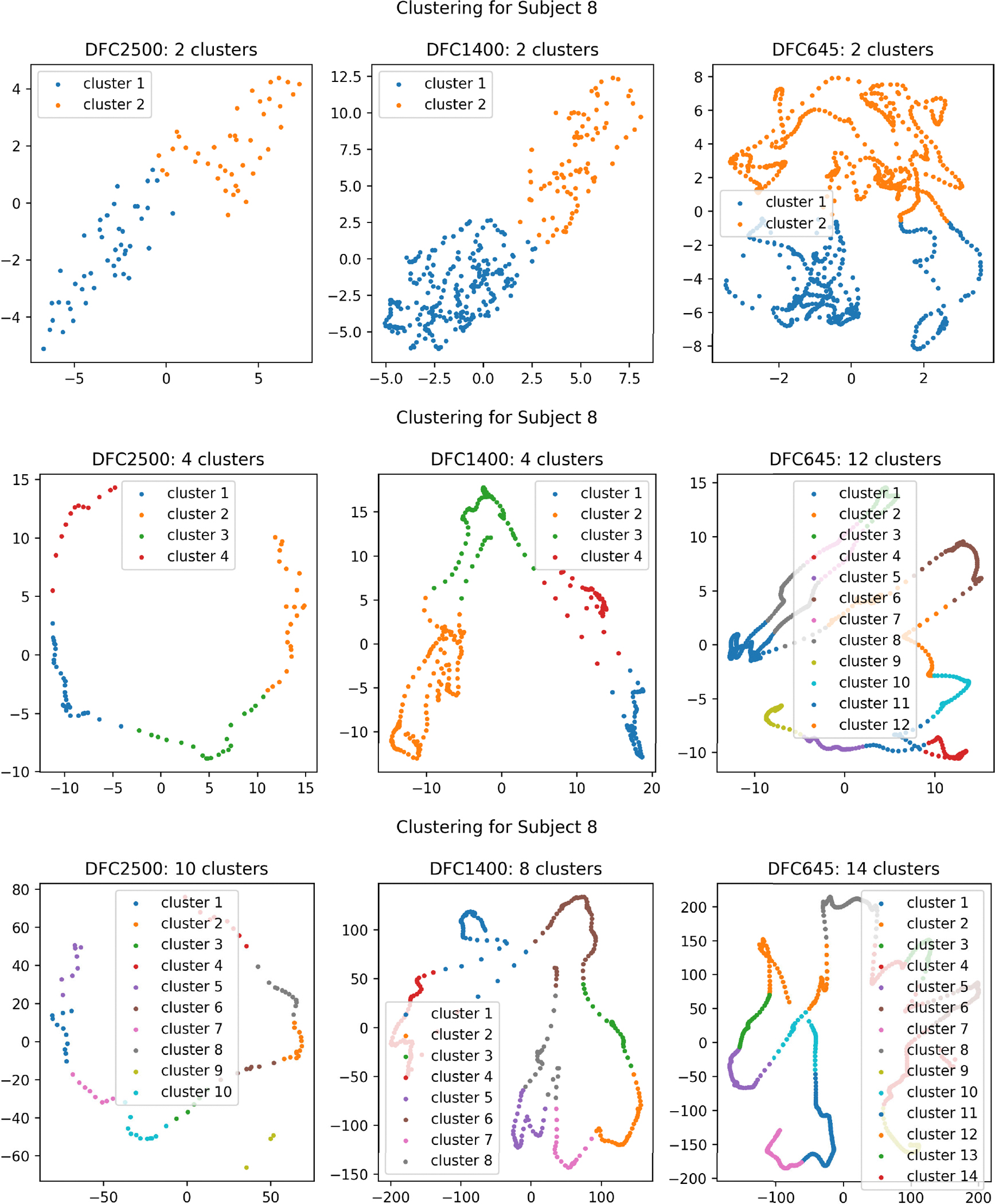
Clustering result for subject 8 across different temporal periods: $f_{\mathrm{low}}=2500ms$ (left), $f_{\mathrm{medium}}=1400ms$ (center), $f_{\mathrm{high}}=645ms$ (right) using TDA-based temporal clustering pipeline (top row). Comparative results for the same subject employing nonTDA-based pipelines, including PCA-based dimensionality reduction and clustering (middle row), and the traditional dFCN clustering pipeline (bottom row).

Figure 7 shows the clustering result for a single subject (subject 8) for all three data cohorts ($f_{\mathrm{low}}=2500ms,\;f_{\mathrm{medium}}=1400ms,\;f_{\mathrm{high}}=645ms$). The top row of the figure represents the plotted clusters using the TDA-based pipeline, and we see that each data cohort here has two clusters. The second row shows the clustering result for same subject using the PCA-based dimensionality reduction and clustering pipeline. While the number of clusters remains consistent for $f_{\mathrm{low}}$ and $f_{\mathrm{medium}}$ in this pipeline, there is a notable discrepancy in the number of clusters for $f_{\mathrm{high}}$. This suggests that the pipeline can not effectively preserve robustness across different data cohorts. The bottom row of the figure shows the plotted clusters for the same subject using the nonTDA-based traditional FCN analysis pipeline, and the number of clusters varies for the data cohorts. As the number of clusters remains unchanged for different temporal sampling periods with similar shape using the TDA-based pipeline and varies largely for the nonTDA-based pipelines, it shows the invariant of the TDA pipeline. We cannot plot the clustering result for direct clustering pipeline due to the elevated size of the data in this clustering analysis (*#timestep*×12, 769), and the absence of any dimensionality reduction techniques (MDS or PCA) applied on the original data. Thus, this illustration gives an intuition towards our hypothesis of the resiliency of persistent homology-based methods to different data acquisition parameters (temporal sampling periods) in brain rs-fMRI data analysis.

To statistically compare the consistency of identified cluster patterns across subjects for both the TDA-based and nonTDA-based pipelines, we compute two similarity metrics - the cohort-wide and pairwise cluster distances. For the cohort-wide analysis, we calculate the absolute difference in number of clusters between each subject’s optimal solution. This provides a distribution of cluster number distances indicating the spread/variability across subjects. For pairwise analysis, we compute cluster distances between each pair of data cohorts for the same subject. The pairwise distances are aggregated to produce a distribution showing the overall pairwise consistency. Lower cohort-wide and pairwise distances indicate higher similarity and consistency in optimal cluster numbers. This quantifies the robustness of each method to individual variations and its ability to extract connectivity patterns that are generalizable across populations. The metrics provide crucial insights into the stability and reproducibility of the clustering solutions.

### A. COHORT-WIDE CLUSTER DISTANCE COMPARISON

We capture the number of clusters for all 316 subjects for all three data cohorts for all the pipelines. We calculate the distance between the number of clusters for each subject using:

(3) $$distance\left({\mathrm{subject}}_{i}\right)=abs\left({subject}_{i_{2500ms}}-{subject}_{i_{1400ms}}\right)\;\;+abs\left({subject}_{i_{1400ms}}-{subject}_{i_{645ms}}\right)\;\;+abs\left({subject}_{i_{645ms}}-{subject}_{i_{2500ms}}\right)$$

where ${subject}_{i_{2500ms}},\;{subject}_{i_{1400ms}},\;{subject}_{i_{645ms}}$ are the number of clusters for ${\mathrm{subject}}_{i}$ for the data cohorts $f_{\mathrm{low}}=2500ms,\;f_{\mathrm{medium}}=1400ms,\;f_{\mathrm{high}}=645ms$ respectively and abs denotes the absolute difference.

**FIGURE 8.**
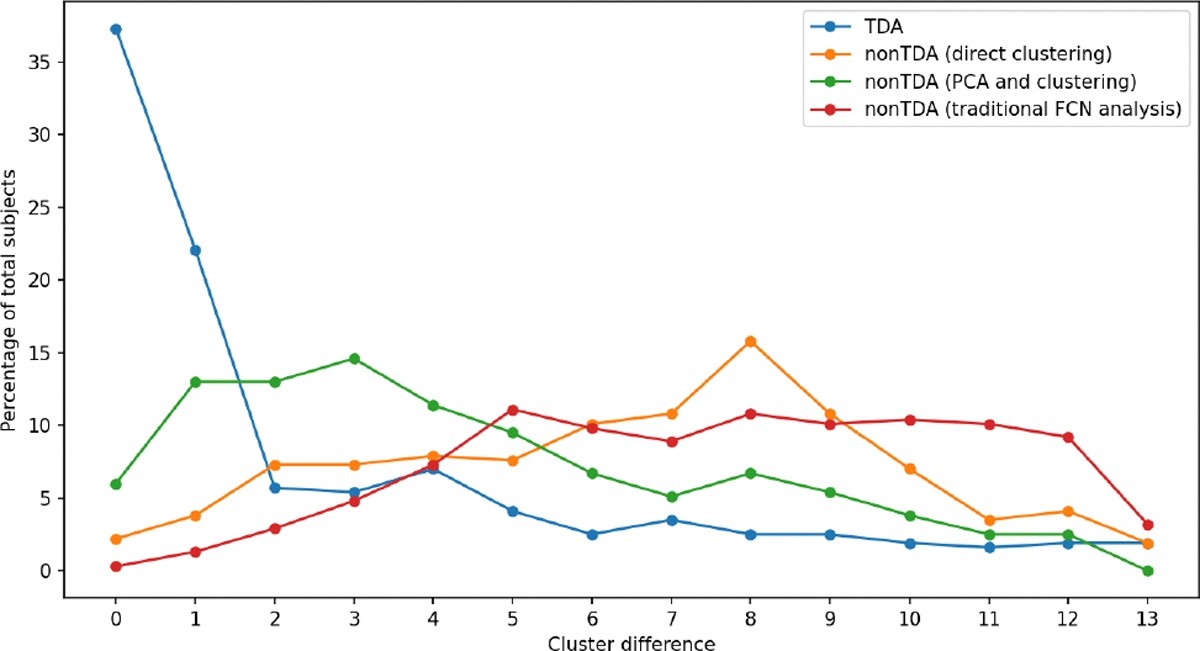
Cohort-wide cluster distance comparison between TDA-based statistical data processing pipeline and nonTDA-based pipelines for all temporal sampling periods $f_{\mathrm{low}}=2500ms,\;f_{\mathrm{medium}}=1400ms$ and $f_{\mathrm{high}}=645ms$. In the TDA-based pipeline, more than 59% of the total subjects exhibit a cluster difference less or equal to 1, indicating the highly robust cluster patterns consistent across cohorts.

The cohort-wide cluster distance analysis in Figure 8 reveals striking differences between the TDA-based and nonTDA-based pipelines. For the TDA pipeline, the majority of subjects (59%) exhibit a tight cluster number distance of less than or equal to one across cohorts. This indicates TDA identifies highly robust cluster patterns consistent across individuals. In contrast, for the direct clustering pipeline, only 6% of subjects have a cohort-wide distance less than or equal to one. For the PCA-based pipeline, this number rises to 19% of subjects and for the traditional FCN analysis plummets to 2% of subjects. The significantly lower consistency highlights the inability of these nonTDA-based techniques to extract stable brain states generalizable across the population. Unlike the topological approach, these methods are heavily influenced by individual variations. Overall, the cohort-wide analysis affirms the resilience of TDA for rs-fMRI analysis, which is able to mitigate differences in data acquisition parameters.

### B. PAIRWISE CLUSTER DISTANCE COMPARISON

**FIGURE 9.**
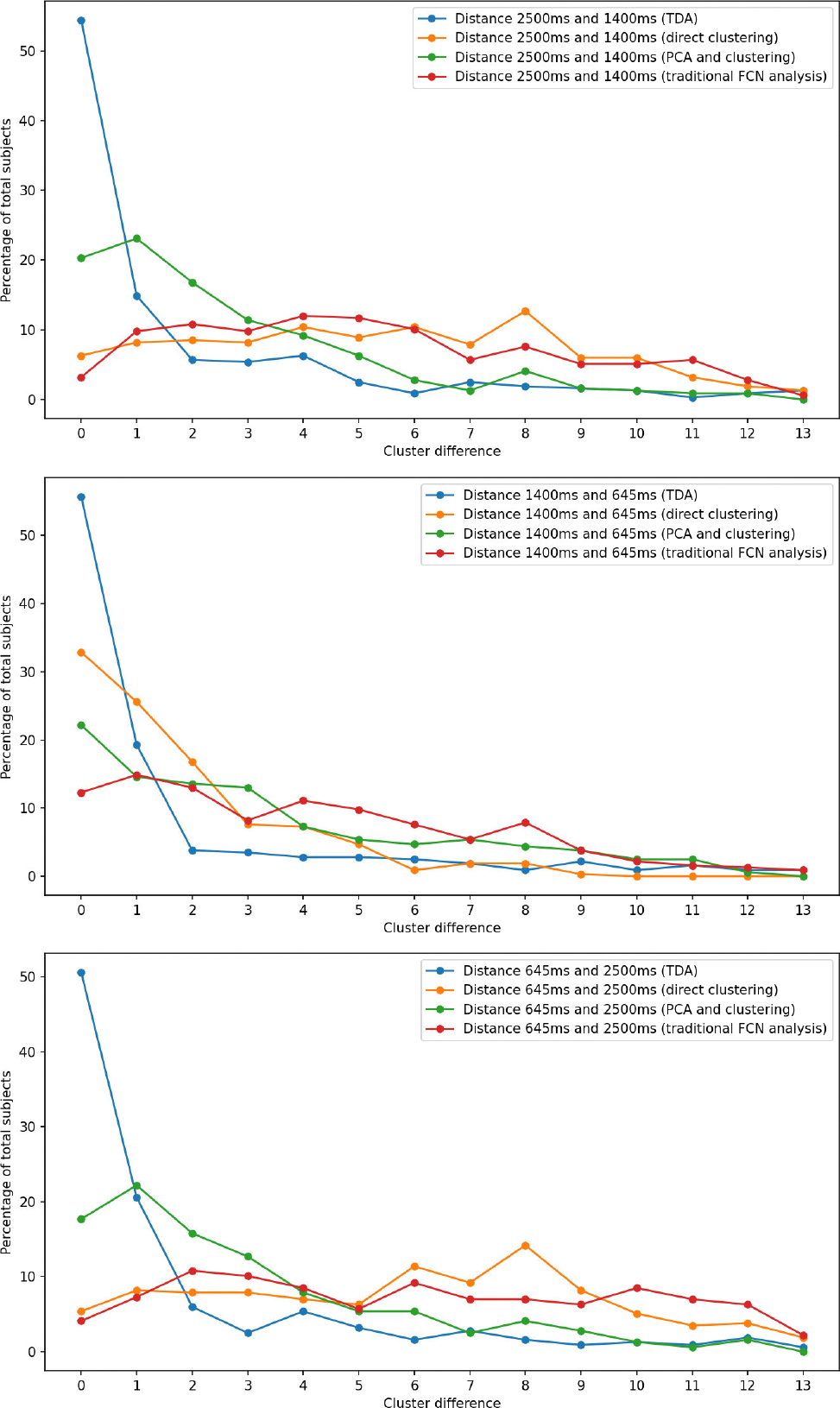
Pairwise cluster distance comparison between TDA-based data processing pipeline and nonTDA-based pipelines. Comparison between temporal sampling $f_{\mathrm{medium}}=1400ms$ and $f_{\mathrm{low}}=2500ms$(top row), temporal sampling $f_{\mathrm{high}}=645ms$ and $f_{\mathrm{medium}}=1400ms$(middle row), temporal sampling $f_{\mathrm{high}}=645ms$ and $f_{\mathrm{low}}=2500ms$(bottom row).

Additionally, we perform a pairwise comparison of the number of clusters for the data cohorts for all of the pipelines. The value of $abs\left({subject}_{i_{2500ms}}-{subject}_{i_{1400ms}}\right)$ represents the pairwise distance on the number of clusters for ${\mathrm{subject}}_{i}$ for the data cohorts $f_{\mathrm{low}}=2500ms$ and $f_{\mathrm{medium}}=1400ms$. Similarly, the value of $abs\left({subject}_{i_{1400ms}}-{subject}_{i_{645ms}}\right)$ and $abs\left({subject}_{i_{645ms}}-{subject}_{i_{2500ms}}\right)$ represent the pairwise distance on the number of clusters between the data cohorts ($f_{\mathrm{medium}}=1400ms,\;f_{\mathrm{high}}=645ms$) and ($f_{\mathrm{high}}=645ms,\;f_{\mathrm{low}}=2500ms$) respectively. This pairwise comparison will help to identify whether there is a closer similarity between the data cohorts in the TDA-based pipeline over the nonTDA-based pipelines. Figure 9 shows the pairwise distance between the data cohorts in the TDA-based pipeline and in the nonTDA-based pipelines. In the TDA pipeline, the pairwise distance between data cohorts $f_{\mathrm{medium}}$ and $f_{\mathrm{high}}$ show the highest similarity (78% matching within distance 2). The data cohort pair $f_{\mathrm{high}}$ and $f_{\mathrm{low}}$ has a similarity of 77% within distance two, and data cohort pair $f_{\mathrm{low}}$ and $f_{\mathrm{medium}}$ has a similarity of 74% within the same distance. This high similarity between the data cohort pairs proves the efficacy of persistent homology-based techniques on the rs-fMRI data analysis with different temporal sampling periods. In the nonTDA-based direct clustering pipeline, we see the maximum similarity between the data cohorts $f_{\mathrm{medium}}$ and $f_{\mathrm{high}}$ with 75% similarity within distance 2. The other data cohort pairs ($\left(f_{\mathrm{low}},f_{\mathrm{medium}}\right)$ and $\left(f_{\mathrm{high}},f_{\mathrm{low}}\right)$) has 23% and 21% similarity within the same distance. In the PCA-based dimensionality reduction and clustering pipeline, we see the maximum similarity between the data cohorts $f_{\mathrm{medium}}$ and $f_{\mathrm{low}}$ with 60% similarity within distance 2. The other data cohort pairs ($\left(f_{\mathrm{medium}},f_{\mathrm{high}}\right)$ and $\left(f_{\mathrm{high}},f_{\mathrm{low}}\right)$) has 50% and 55% similarity within the same distance. In the last nonTDA-based traditional FCN analysis pipeline, we see the maximum similarity between the data cohorts $f_{\mathrm{medium}}$ and $f_{\mathrm{high}}$ with 40% similarity within distance 2. The other data cohort pairs ($\left(f_{\mathrm{low}},f_{\mathrm{medium}}\right)$ and $\left(f_{\mathrm{high}},f_{\mathrm{low}}\right)$) has 23% and 22% similarity within the same distance. This low similarity between the data cohorts using nonTDA-based pipelines indicates the inefficiency of the nonTDA-based method for analysing rs-fMRI data with different data acquisition parameters.

### C. EVALUATION USING A CLINICAL ADHD DATASET

**FIGURE 10.**
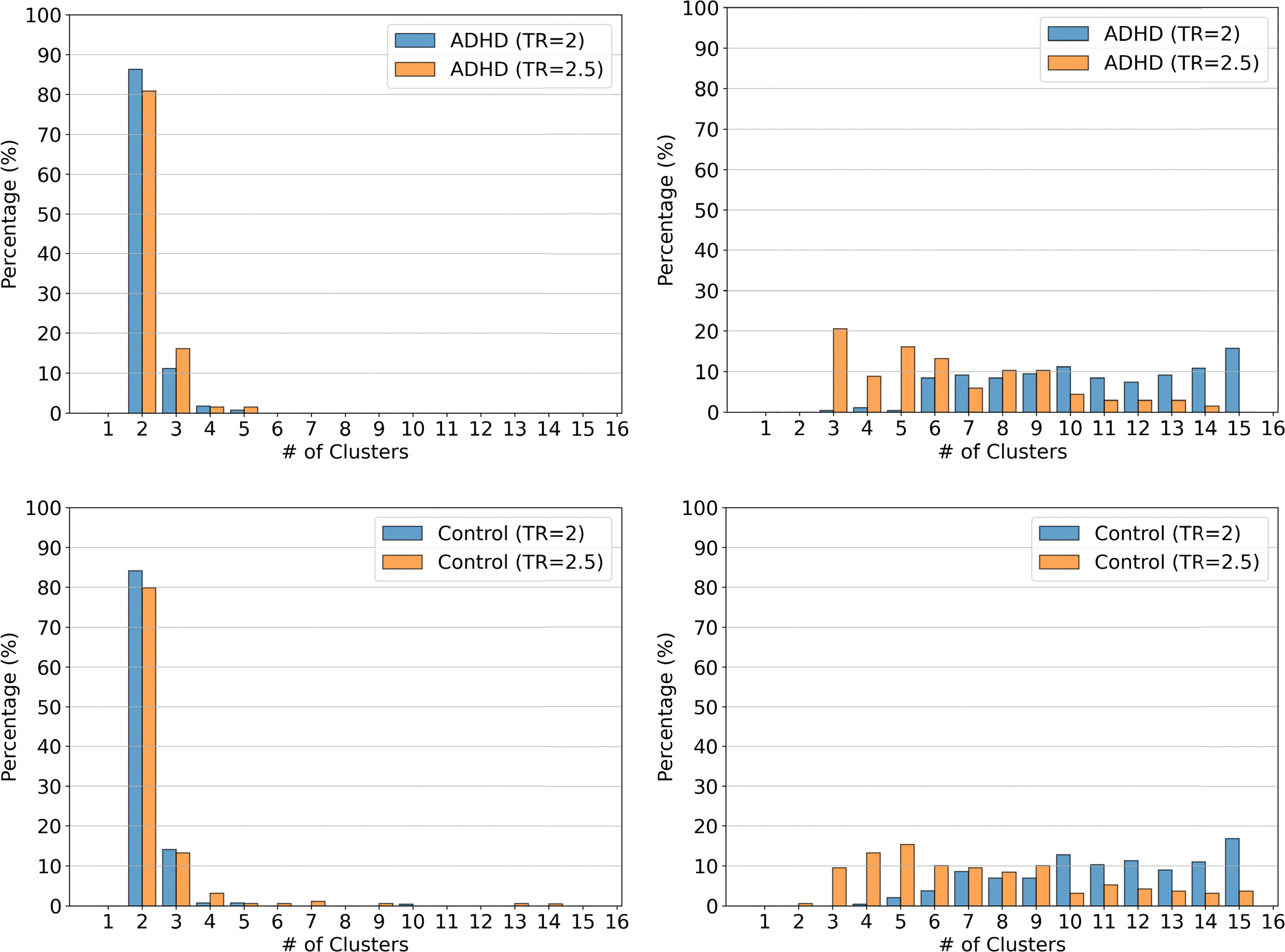
Comparison of the number of clusters obtained using TDA-based pipeline (left column) vs traditional dFCN pipeline (right column) for ADHD (top row) and Control (bottom row) groups across TR=2s and TR=2.5s. The TDA pipeline shows high consistency reflected by higher similarity (≥ 80%) in the number of identified clusters. The nonTDA-based traditional dFCN pipeline exhibits variability and sensitivity to TR differences.

To validate the robustness of our TDA-based temporal clustering pipeline, we conducted a comparative evaluation against the traditional dynamic functional connectivity network (dFCN) pipeline using the publicly available ADHD-200 dataset [63], [64]. We constructed two cohorts based on the temporal resolution (TR) of the rs-fMRI scans: TR=2s and TR=2.5s. Each cohort comprises ADHD and control subjects. The TR=2s cohort includes 290 control and 285 ADHD subjects, while the TR=2.5s cohort consists of 189 control and 68 ADHD subjects. Figure 10 presents a systematic comparison of clustering consistency between the TDA-based pipeline (left column) and traditional dFCN pipeline (right column) across ADHD and control groups. Each subplot shows the distribution of the number of clusters across all subjects within a group and TR condition. The x-axis denotes the number of clusters (ranging from 1 to 16), and the y-axis indicates the percentage of subjects exhibiting each cluster count.

In the TDA-based pipeline (left column), we observe a strong peak at 2 clusters for both ADHD and control subjects across TR=2s and TR=2.5s. This suggests a high degree of consistency in the extracted brain state patterns, with over 80% of subjects in each subgroup consistently showing two clusters. This invariance across different TRs demonstrates the robustness of persistent homology and topological features in summarizing the intrinsic structure of time-varying brain connectivity.

In contrast, the traditional dFCN pipeline (right column) exhibits significant variability in the number of clusters across TRs. For both ADHD and control groups, the cluster distributions are dispersed, with subjects assigned to a wide range of cluster numbers, especially at higher TRs. For instance, in the ADHD group (top right), the cluster counts span from 3 to 15, with no dominant mode. This inconsistency indicates the sensitivity of the dFCN pipeline to changes in temporal resolution and its reduced ability to extract reproducible brain state signatures.

This analysis highlights the advantage of using topological features derived from persistent homology over traditional correlation-based approaches. While dFCN pipelines are prone to capturing noise and suffer from over-fragmentation of temporal brain states, the TDA pipeline produces stable and interpretable cluster structures. These findings support the hypothesis that TDA provides a more reliable abstraction of temporal dynamics in brain connectivity, particularly in clinical neuroimaging datasets with acquisition variability.

### D. COMPUTATIONAL COMPLEXITY ANALYSIS

We now analyze the computational complexity of the TDA-based (Sec. III) and nonTDA-based pipelines (Sec. IV) up to the statistical analysis stage. Since the statistical analysis stage is common across all pipelines, our analysis focuses on the pipeline-specific processing steps prior to statistical testing. The computational trade-offs of the pipelines are summarized in Table 1.

Let S denote the number of subjects (S=316),T the number of timepoints per subject (T=754 in the largest case), D the number of regions of interest (D=113), and $D^{2}=12,\;769$ the number of features in each flattened functional connectivity network (FCN). The number of clusters explored in KMeans is k=15, and the maximum number of KMeans iterations is $T_{k}=300$.

#### 1) TDA-BASED PIPELINE

In the TDA-based pipeline, each subject and timepoint undergoes persistent homology computation on its FCN, resulting in a total cost of $S\cdot T\cdot C_{ph}$. The computational cost for generating a single persistence barcode is denoted by $C_{ph}$. For our approach, using the Gudhi library with the Vietoris–Rips complex and max_dimension=1 on D=113 ROIs, the time complexity for 0-dimensional persistent homology scales as $C_{ph}=O\left(D^{2}log\;D\right)$, which yields approximately 86,800 operations per timepoint. After barcode extraction, each subject requires a pairwise Wasserstein distance matrix between all timepoints ($S\cdot T^{2}$), two-dimensional MDS on this matrix ($S\cdot T^{2}$), and k-means clustering ($S\cdot T\cdot k\cdot T_{k}$). The total computational complexity is:

$$𝒪\left(S\cdot T\cdot D^{2}log\;D+S\cdot T^{2}+S\cdot T\cdot k\cdot T_{k}\right)$$

which, for the largest case, evaluates to 20,871 M.

#### 2) DIRECT CLUSTERING PIPELINE

For direct clustering, all FCNs for a subject are flattened into a ($T\times D^{2}$) matrix, and k-means clustering is applied in the high-dimensional space. The complexity is:

$$𝒪\left(S\cdot T\cdot D^{2}\cdot k\cdot T_{k}\right)$$

which evaluates to 13,695,004 M.

#### 3) PCA-BASED PIPELINE

In the PCA-based pipeline, the ($T\times D^{2}$) FCN matrix is first reduced to two dimensions using PCA, which has complexity $S\cdot T\cdot {\left(D^{2}\right)}^{2}=S\cdot T\cdot D^{4}$ per subject (due to SVD on high-dimensional data), followed by k-means clustering on the reduced matrix. The total computational complexity is:

$$𝒪\left(S\cdot T\cdot D^{4}+S\cdot T\cdot k\cdot T_{k}\right)$$

which for the largest case evaluates to 38,860, 145 M.

#### 4) TRADITIONAL DFCN CLUSTERING PIPELINE

In the traditional dFCN pipeline, for each subject we compute a pairwise Euclidean distance matrix (T×T) between all D×D adjacency matrices at all timepoints, with a cost of $S\cdot T^{2}\cdot D^{2}$. This is followed by the same $S\cdot T^{2}$ MDS embedding and $S\cdot T\cdot k\cdot T_{k}$ k-means clustering as in the TDA-based pipeline. The total computational complexity is:

$$𝒪\left(S\cdot T^{2}\cdot D^{2}+S\cdot T^{2}+S\cdot T\cdot k\cdot T_{k}\right)$$

which, for the largest case, is 2,290, 525 M.

### E. COMPARISON BETWEEN TDA-BASED AND NON-TDA BASED PIPELINES

We extensively evaluated the accuracy and robustness of our TDA pipeline for rs-fMRI analysis on a large-scale healthy control dataset comprising 371,616 adjacency matrices across 316 subjects as well as a clinical ADHD dataset comprising of 832 subjects. Comparisons are made to three alternative approaches - direct clustering, PCA, and traditional FCN analysis.

Table 2 summarizes the systematic comparison between TDA-based and NonTDA-based data processing pipelines in terms of methodology, dimensionality reduction, cluster interpretability, robustness (cohort-wide and pairwise similarities), as well as their advantages and limitations. Clearly, the TDA-based pipeline demonstrates significantly higher robustness, interpretability, and consistency across different temporal data acquisition parameters, albeit with higher computational overhead, when compared to the NonTDA-based methods. The NonTDA-based pipelines vary in their advantages–such as simplicity, computational efficiency, and low computational cost–but generally exhibit reduced robustness, higher noise sensitivity, and lower interpretability. The results demonstrate TDA’s superior ability to extract robust and invariant topological signatures intrinsically linked to resting-state functional architectures. Remarkably, the brain states identified by TDA exhibit high consistency across the three sampling frequencies, affirming resilience to acquisition variations. This also highlights TDA’s efficacy in mitigating non-neural variability and capturing fundamental dynamics as compared to conventional techniques. By applying the persistent homology technique to filter noise and reveal salient connectivity motifs, TDA provides a principled graph-free technique for preserving temporal dynamics of complex rs-fMRI data. These findings establish persistent homology as a powerful approach for analyzing temporal patterns and validating TDA as a promising pipeline for robust discovery of data-driven functional brain states.

## VI. DISCUSSION

MRI scanners around the globe vary in their configurations and field strengths. This variation leads to a certain level of noise in the data collected due to non-neural differences introduced by the diverse scanner setups and data collection parameters. This noise complicates the process of combining data from different scanners into a single, large dataset for unified analysis. Consequently, most fMRI brain network research is localized, limited by the number of subjects that can be scanned at a single location. This limitation reduces the sample size and, therefore, the applicability of the results. One solution is to conduct studies across multiple sites closer to the target population. However, the noise introduced by using different scanners and parameters diminishes the neural effects of interest, thereby reducing the effectiveness of such multi-site efforts.

We addressed these issues in our previous paper [8] in the context of characterizing brain networks using static functional connectivity. However, DFC is critical for understanding how the brain processes information dynamically and how the interactions between different brain regions change with time. It has been shown that DFC is very important for characterizing the healthy brain [3], [4], as well as in various brain disorders [5], [6], [7]. Therefore, it becomes necessary to develop a TDA-based framework for DFC so that investigations of temporal dynamics in the brain are shielded from non-neural variability in the data.

We validated the effectiveness of the proposed Topological Data Analysis (TDA)-based pipeline by contrasting it with the conventional data analysis pipelines outlined in Section IV. In the conventional pipelines, we employed direct time-series clustering, PCA-based dimensionality reduction and clustering, as well as traditional dynamic FCN pipeline with MDS-based dimensionality reduction. The outcomes of this pipeline strongly suggest that these conventional methods fail to establish similarity across dynamic FCNs of the same subjects obtained with different repetition times (TRs) and acquisition parameters.

On the contrary, for the TDA-based metric, we demonstrated both qualitatively and quantitatively that the metric remains statistically consistent across the same subjects, regardless of the sampling period used to acquire resting-state fMRI data. This underscores the usefulness of TDA-based analysis because, theoretically, data collected using different parameters from the same subject should still represent the same brain network dynamics.

## VII. CONCLUSION

In this study, we have demonstrated the effectiveness of Topological Data Analysis (TDA) in uncovering temporal properties within resting-state functional magnetic resonance imaging (rs-fMRI) data. Our research highlights TDA’s robustness in the presence of varying temporal sampling rates, surpassing traditional connectivity analysis methods. Key findings emphasize TDA’s remarkable stability, with 59% of subjects consistently showing clustering results across different temporal sampling periods (2500*ms*, 1400*ms*, 645*ms*), compared to less than 19% using nonTDA-based methods. TDA also reveals strong pairwise similarities between sampling periods, showcasing its ability to capture temporal dynamics. The robustness of the TDA pipeline is further confirmed through evaluation on clinical ADHD datasets, where it achieves high consistency (≥ 80%) in clustering outcomes across different sites and scanning conditions. In conclusion, our study establishes TDA as a valuable tool for revealing temporal nuances in rs-fMRI data, offering a level of robustness unmatched by traditional methods. Through persistent homology, TDA provides a stable, invariant representation of dynamic brain connectivity, promising valuable insights into complex temporal patterns in resting-state fMRI data across diverse acquisition parameters. To promote reproducibility, we have made all our code, scripts, data, and documentation available at https://github.com/harp-lab/TemporalBrainPH.

## Figures and Tables

**TABLE 1.** Computational trade-offs of each pipeline up to the statistical analysis stage. The second column shows the general Big-O notation, while the third column gives the evaluated value in millions (M) for the largest values of the variables denoting *S* = 316, *T* = 754, *D* = 113 (*D*^2^ = 12,769), *k* = 15, *T_k_* = 300. *Example: For TDA-based: for the largest case, evaluates to* 316 · 754 · 86,800 + 316 · 754^2^ + 316 · 754 · 15 · 300 = 20,871 M.

Pipeline	Computational Complexity (Big-O)	Evaluated value (in M)
TDA-based	$𝒪\left(S\cdot T\cdot C_{ph}+S\cdot T^{2}+S\cdot T\cdot k\cdot T_{k}\right)$	20,871 M
Direct clustering	$𝒪\left(S\cdot T\cdot D^{2}\cdot k\cdot T_{k}\right)$	13,695,004 M
PCA-based	$𝒪\left(S\cdot T\cdot D^{4}+S\cdot T\cdot k\cdot T_{k}\right)$	38,860,145 M
Traditional dFCN	$𝒪\left(S\cdot T^{2}\cdot D^{2}+S\cdot T^{2}+S\cdot T\cdot k\cdot T_{k}\right)$	2,290,525 M

**TABLE 2.** Systematic comparison between TDA-based and NonTDA-based data processing pipelines for rs-fMRI dataset.

Criteria	TDA-based pipeline	NonTDA-based pipelines		
		Direct clustering	PCA pipeline	Traditional dFCN
Methodology	Persistent homology + clustering	Direct clustering	PCA + clustering	Correlation + clustering
Dimensionality reduction (Distance function)	MDS (Wasserstein distance)	None	PCA	MDS (Euclidean distance)
Cluster interpretability (Cohort-wide similarity)	High (59%)	Low (6%)	Low (19%)	Low (2%)
Robustness (Pairwise similarity)	High (78%, 77%, 74%)	Moderate (75%, 23%, 21%)	Moderate (60%, 55%, 50%)	Low (40%, 23%, 22%)
Advantages	High robustness; Effective noise filtering; Clear interpretability	Simple, straightforward; No preprocessing required	Moderate robustness; Reduced dimensions	Low computational cost; Reduced dimensions
Limitations	Additional computations	High dimensionality; Noise sensitive; Poor interpretability	Poor interpretability; Moderate robustness	Poor interpretability; Noise sensitive
